# Bacteria associated with Zn-hyperaccumulators *Arabidopsis halleri* and *Arabidopsis arenosa* from Zn–Pb–Cd waste heaps in Poland as promising tools for bioremediation

**DOI:** 10.1038/s41598-023-39852-6

**Published:** 2023-08-03

**Authors:** Ewa Oleńska, Wanda Małek, Małgorzata Wójcik, Sebastian Szopa, Izabela Swiecicka, Olgierd Aleksandrowicz, Tadeusz Włostowski, Weronika Zawadzka, Wouter M. A. Sillen, Jaco Vangronsveld, Iva Cholakova, Tori Langill, Sofie Thijs

**Affiliations:** 1 Faculty of Biology, University of Bialystok, 1J Ciołkowski, 15-245 Bialystok, Poland; 2 Faculty of Biology and Biotechnology, Institute of Biological Sciences, Maria Curie-Skłodowska University, 19 Akademicka, 20-033 Lublin, Poland; 3 SHIM-POL A.M. Borzymowski, 5 Lubomirski, 05-080 Izabelin, Poland; 4 Laboratory of Applied Microbiology, University of Bialystok, 1J Ciołkowski, 15-245 Bialystok, Poland; 5 Faculty of Environmental Biology, Centre for Environmental Sciences, Hasselt University, Agoralaan Building D, 3590 Diepenbeek, Belgium

**Keywords:** Applied microbiology, Microbial communities, Environmental microbiology

## Abstract

To identify metal adapted bacteria equipped with traits positively influencing the growth of two hyperaccumulator plant species *Arabidopsis arenosa* and *Arabidopsis halleri*, we isolated bacteria inhabiting rhizosphere and vegetative tissues (roots, basal and stem leaves) of plants growing on two old Zn–Pb–Cd waste heaps in Bolesław and Bukowno (S. Poland), and characterized their potential plant growth promoting (PGP) traits as well as determined metal concentrations in rhizosphere and plant tissues. To determine taxonomic position of 144 bacterial isolates, 16S rDNA Sanger sequencing was used. A metabolic characterization of isolated strains was performed in vitro using PGP tests. *A. arenosa* and *A. halleri* accumulate high amounts of Zn in their tissues, especially in stem leaves. Among in total 22 identified bacterial taxa, the highest level of the taxonomical diversity (*H’* = 2.01) was revealed in *A. halleri* basal leaf endophytes originating from Bukowno waste heap area. The 96, 98, 99, and 98% of investigated strains showed tolerant to Cd, Zn, Pb and Cu, respectively. Generally, higher percentages of bacteria could synthesize auxins, siderophores, and acetoin as well as could solubilize phosphate. Nine of waste heap origin bacterial strains were tolerant to toxic metals, showed in vitro PGP traits and are potential candidates for bioremediation.

## Introduction

Anthropogenic activities related release of metals has strongly contributed to environmental pollution on a wide world scale^1^. Metals as non-degradable elements can only be transformed into chemical forms of altered toxicity and/or mobility. They can enter into food chains and cause toxicity to organisms^2^. Implementation of diverse physical methods of remediation such as soil excavation and land filling, soil washing, electroremediation, vitrification, and chemical treatments such as precipitation, leaching, extraction, ion exchange, encapsulation or immobilization usually result in a reduction of metal reactivity but often produce byproducts^3,4^. Physicochemical remediation methods have negative effects on microbial life as well as on several soil parameters, like pH, clay and organic matter^5,6^. They also cannot be applied at large scale since they are generally too expensive, and their public acceptace is low^3^. As an alternative, plant-based strategies (often termed ‘phytoremediation’) have been proposed as more eco-friendly methods for the restoration of degraded soils^7–9^. Plants that avoid metal toxicity by storing metal ions in their underground tissues are used as phytostabilizers, whereas plants that accumulate metals in aboveground tissues can be applied as phytoextractors^10^. When a phytoextractor plant is taking up metals from soils and reaches certain threshold leaf metal concentrations it is classified as hyperaccumulator^11^. Actually, more than 500 metal hyperaccumulators have been described; a majority of them are obligate metallophytes restricted to metalliferous soils, whereas a smaller group involves facultative hyperaccumulators inhabiting both non-metalliferous and metalliferous soils^12,13^. In spite of an extensive understanding about the mechanisms of adaptation of metallophytes, it is clear that using such plants only for phytoextraction is not economically feasible because of their generally low growth rate and limited biomass production^14,15^. It has been recently shown that a combined use of plants with specific microorganisms may substantially increase the efficiency of remediation, for both organic pollutants and metals^16–19^. Plant endophytes can alleviate various types of stresses, e.g. salinity^20^, drought^21^, osmotic stress^22^, temperature^23^, and metal toxicity^24–27^. Sánchez-López et al.^28^, for example, demonstrated that the metal tolerant *Methylobacterium* sp. strain Cp3 isolated from seeds of *Crotolaria pumila* growing on Zn-polluted soil showed in in vitro tests multiple traits that can have beneficial effect on plant growth and thus can be potentially useful in phytoremediation. Microbes under metal stress conditions can interact with ions directly^29–31^ or can beneficially influence the fitness of their host plants including those that accumulate toxic metals in their tissues, reducing metal toxicity^32–35^. The knowledge about bacterial strains potentially useful in remediation of metal polluted soils is of significant importance.

The about 100-yrs old waste heaps in Bolesław and Bukowno in Southern Poland are post-mining deposits containing high metal concentrations (50,000 and 20,159 mg Zn kg^−1^; 5000 and 35 mg Pb kg^−1^; 500 and 18 mg Cd kg^−1^ respectively)^36^, that exceed to a significant extent the permissible metal concentrations proposed by WHO: 50 mg Zn kg^−1^, 85 mg Pb kg^−1^, 0.8 mg Cd kg^−1^^37–39^. They harbor quite a number of plants and bacteria that adapted to metal toxicity^40–43^. For example, *Arabidopsis halleri* (L.) O’Kane and Al-Shehbaz (formerly *Cardaminopsis halleri* Hayek s. l.) and closely related *Arabidopsis arenosa* (L.) Lawalrée (formerly *Cardaminopsis arenosa* (L.) Hayek s. l.) along with associated microorganisms are natural inhabitants of these waste heaps. *A halleri* is an thoroughly investigated hyperaccumulator of Zn^44–49^, whereas *A. arenosa* (sand rock-cress) is recently recognized as hyperaccumulator of Zn and Cd^50^. It was shown that endophytes of *Trifolium repens* L., inhabiting calamine waste heaps in Southern Poland, contribute significantly to the phytostabilization potential of this plant species^36,51,52^. Significantly more white clover endophytes of the Zn–Pb–Cd waste heap origin than of the reference one revealed as metal tolerant an able to synthesize ACC-deaminase, acetoin, and siderophores. Moreover, two strains, *Bacillus megaterium* BolR EW3_A03 and *Stenotrophomonas maltophilia* BolN EW3_B03 were proposed as promising for application in phytostabilization of Zn, Pb or Cd polluted areas^36^. Therefore, it sounds likely that the hyperaccumulators *A. halleri* and *A. arenosa,* growing on about 100-yrs old waste heaps in Southern Poland, are natural reservoirs of adapted bacterial strains equipped with traits that can be beneficial to plants that might have potential for application as a tool in remediation. In order to identify metal adapted bacterial strains equipped with potential plant growth promoting traits, we studied the metal concentrations in soils and plants and the bacteria isolated from the rhizosphere and different parts (roots, rosette/basal leaves, and stem leaves) of *A. halleri* and *A. arenosa* growing on the above-mentioned Zn–Pb–Cd polluted waste heaps in Bolesław and Bukowno. The taxonomic position of bacterial isolates was determined using 16S rRNA gene Sanger sequencing. Also their in vitro potential for synthesizing siderophores, organic acids, acetoin, and indole-3-acetic acid (IAA, auxin), the activity of 1-aminocyclopropane-1-carboxylate (ACC)-deaminase (ACCD), and their ability for phosphate solubilization and fixation of atmospheric nitrogen were examined.

## Results

The examination of 144 cultivable bacterial strains isolated from vegetative tissues and rhizosphere of *A. arenosa* and *A. halleri* revealed significant differences in bacterial species diversity depending on host plant species, plant habitat and type of the tissue. For *A. arenosa,* the highest value of strain diversity index (*ISD* = 100%) was found for the endophytes of stem leaves of plants growing on the Bolestraszyce reference area, while the lowest one (23%) was found for *A. arenosa* root endophytes from the Bolesław area (Table 1). For *A. halleri* endophytes, the highest value of strain diversity index (89%) was noticed in rosette leaves of plants growing on the Bukowno waste heap, the lowest one (50%) in rosette leaves of plants originating from the Bolesław waste heap (Table 1). The index of strain diversity of studied *A. arenosa* and *A. halleri* rhizosphere communities, except *A. arenosa* rhizosphere bacteria of Bukowno origin (80%), was 100%. The Shannon’s diversity index (*H*’) of the *A. arenosa* endophyte community was the highest in the rosette leaves from Bukowno (*H*’ = 1.67), while the lowest level (*H*’ = 0) was detected for the stem leaf endophytic community from the reference Bolestraszyce area (Table 1). Both *A. halleri* rhizosphere bacterial communities showed significantly lower levels of strain richness (*H’* = 1.09) compared to the *A. arenosa* reference and waste heap communities (Table 1).

**Table 1:** Index of strain diversity (*ISD*) and Shannon’s diversity index (*H’*) indices in endophytic bacterial populations of *A. halleri* and *A. arenosa* roots (R), rosette leaves (RL), stem leaves (SL), and rhizosphere (RS) of the waste heap Bolesław (BOL) and Bukowno (BUK) origin as well as the reference Bolestraszyce (BCE) area.

	*ISD*	*H’*
*A. arenosa* R, BOL	23	0.92
*A. arenosa* RL, BOL	46	1.54
*A. arenosa* SL, BOL	37	1.63
*A. arenosa* RS, BOL	100	1.39
*A. arenosa* R, BUK	80	1.33
*A. arenosa* RL, BUK	75	1.67
*A. arenosa* SL, BUK	67	1.18
*A. arenosa* RS, BUK	80	1.32
*A. arenosa* R, BCE	56	1.47
*A. arenosa* RL, BCE	60	0.95
*A. arenosa* SL, BCE	100	0
*A. arenosa* RS, BCE	100	1.78
*A. halleri* R, BOL	80	1.32
*A. halleri* RL, BOL	50	0.87
*A. halleri* SL, BOL	56	1.50
*A. halleri* RS, BOL	100	1.09
*A. halleri* R, BUK	63	1.5
*A. halleri* RL, BUK	89	2.01
*A. halleri* SL, BUK	67	1.32
*A. halleri* RS, BUK	100	1.09

**Figure 1:**
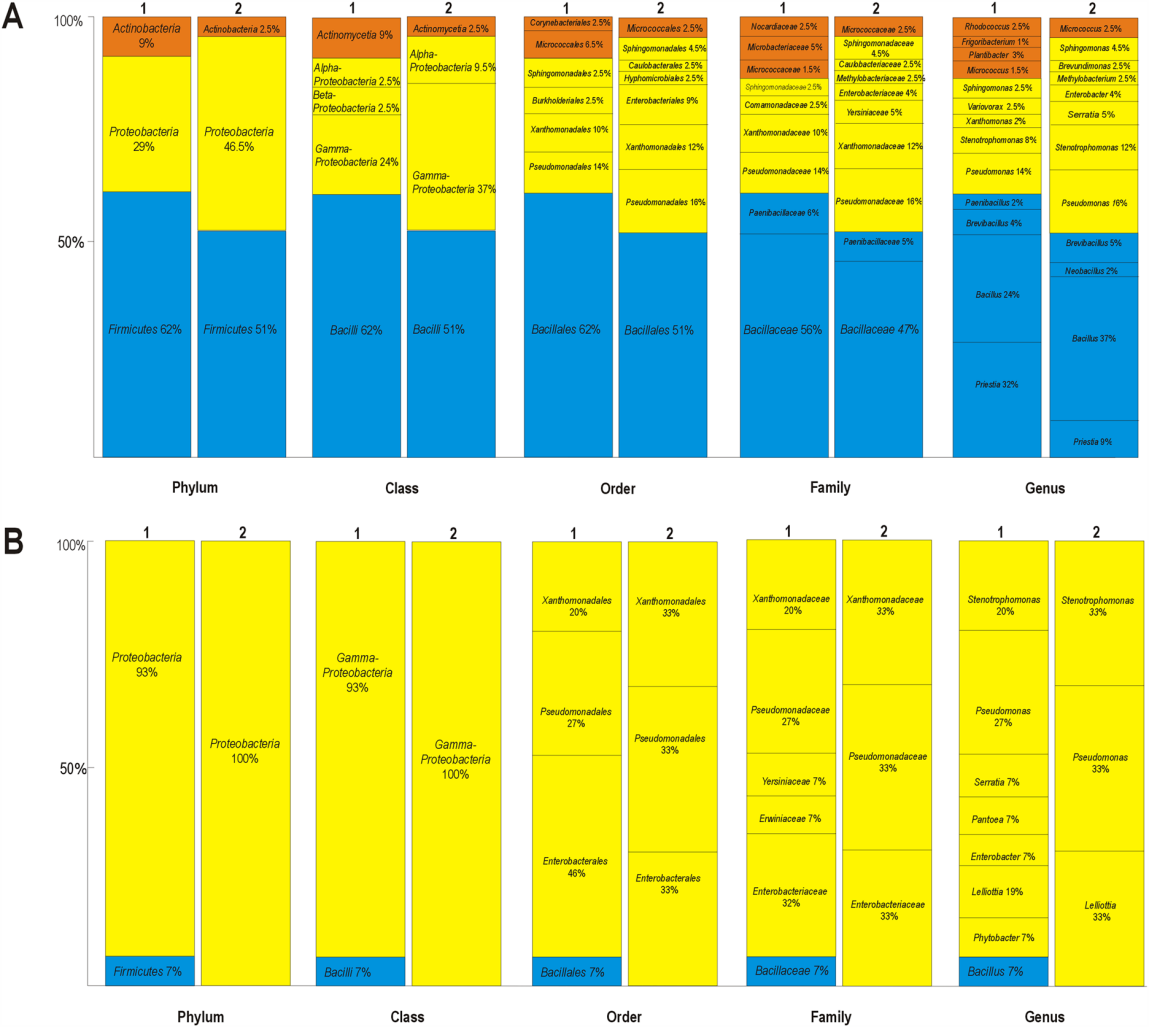
Taxonomy of bacterial strains izolated from stem leaves, rosette leaves, and roots (**A**) as well as rhizosphere (**B**) of *A. arenosa* (1) and *A. halleri* (2) growing on the Zn–Pb–Cd waste heaps and reference area.

16S rRNA gene analysis revealed the diversity of the endophytic and rhizosphere microorganisms of *A. arenosa* and *A. halleri*. 62 and 51% of the endophytes of *A. arenosa* and *A. halleri* belonged to *Firmicutes*, 29 and 46.5% to *Proteobacteria*, and 9 and 2.5% to *Actinobacteria*, respectively (Fig. 1A). 62% of the endophytic strains of *A. arenosa* were *Bacilli*, the classes *α-* and *β-Proteobacteria* were represented by approximately 2.5% of the isolated strains, the *γ-Proteobacteria* by 24%, and the *Actinomycetia* by 9%. 51% of the isolated endophytes from *A halleri* were classified as *Bacilli*, 37% *γ-Proteobacteria*, 9.5% *α-Proteobacteria*, and 2.5% *Actinomycetia*. In both, *A. arenosa* and *A. halleri*, the *Bacillales* were found to be the most abundant order, the least abundant in *A. arenosa* were *Burkholderiales* (2.5%), *Sphingomonadales* (2.5%), and *Corynebacteriales* (2.5%), whereas in *A. halleri* the members of *Caulobacterales* (2.5%), *Hyphomicrobiales* (2.5%), and *Micrococcales* (2.5%) showed the lowest abundancies. Bacteria belonging to the genera *Priestia* sp. (32%), *Bacillus* sp. (24%), and *Pseudomonas* sp. (14%) were found as dominant endophytes of *A. arenosa*, while in *A. halleri* the prevailing bacterial endophytes were *Bacillus* sp. (37%), *Pseudomonas* sp. (16%), and *Stenotrophomonas* sp. (12%) (Fig. 1A).

Among 21 bacterial strains isolated from the rhizosphere of *A. arenosa* (15 isolates) and *A. halleri* (6 isolates) 95% of them were *Proteobacteria*, while 5% were *Firmicutes*. The members of the class *Bacilli*, represented by 5% of the isolated rhizosphere bacteria were members of *Bacillales*, and *Bacillaceae*. Almost 95% of the *γ-Proteobacteria* belonged to the orders *Pseudomonadales* (29%), *Enterobacterales* (43%), and *Xanthomonadales* (23%), and were members of the families *Pseudomonadaceae* (29%), *Enterobacteriaceae* (33%), *Xanthomonadaceae* (23%), *Erwiniaceae* (5%), and *Yersiniaceae* (5%). *A. arenosa* endophytes consisted of *γ-Proteobacteria* (93%) and *Firmicutes* (7%) (Fig. 1B). The *γ-Proteobacteria* from the rhizosphere of *A. arenosa* belonged to the orders *Enterobacterales* (46%), *Pseudomonadales* (27%), and *Xanthomonadales* (20%), and families: *Enterobacteriaceae* (32%), *Pseudomonadaceae* (27%), *Xanthomonadaceae* (20%), *Yersiniaceae* (7%), and *Erwiniaceae* (7%), while the strains belonging to the *Bacilli* class (7% of the rhizosphere inhabitants) were members of *Bacillales*, and family of *Bacillaceae*. All cultivable bacterial inhabitants of the rhizosphere of *A. halleri* belonged to the class *γ-Proteobacteria* (Fig. 1B). 33% of them were members of the order *Enterobacterales* and family *Enterobacteriaceae*, 33% *Pseudomonadales* and *Pseudomonadaceae*, and 33% were members of order *Xanthomonadales* and family *Xanthomonadaceae* (Fig. 1B).

**Figure 2:**
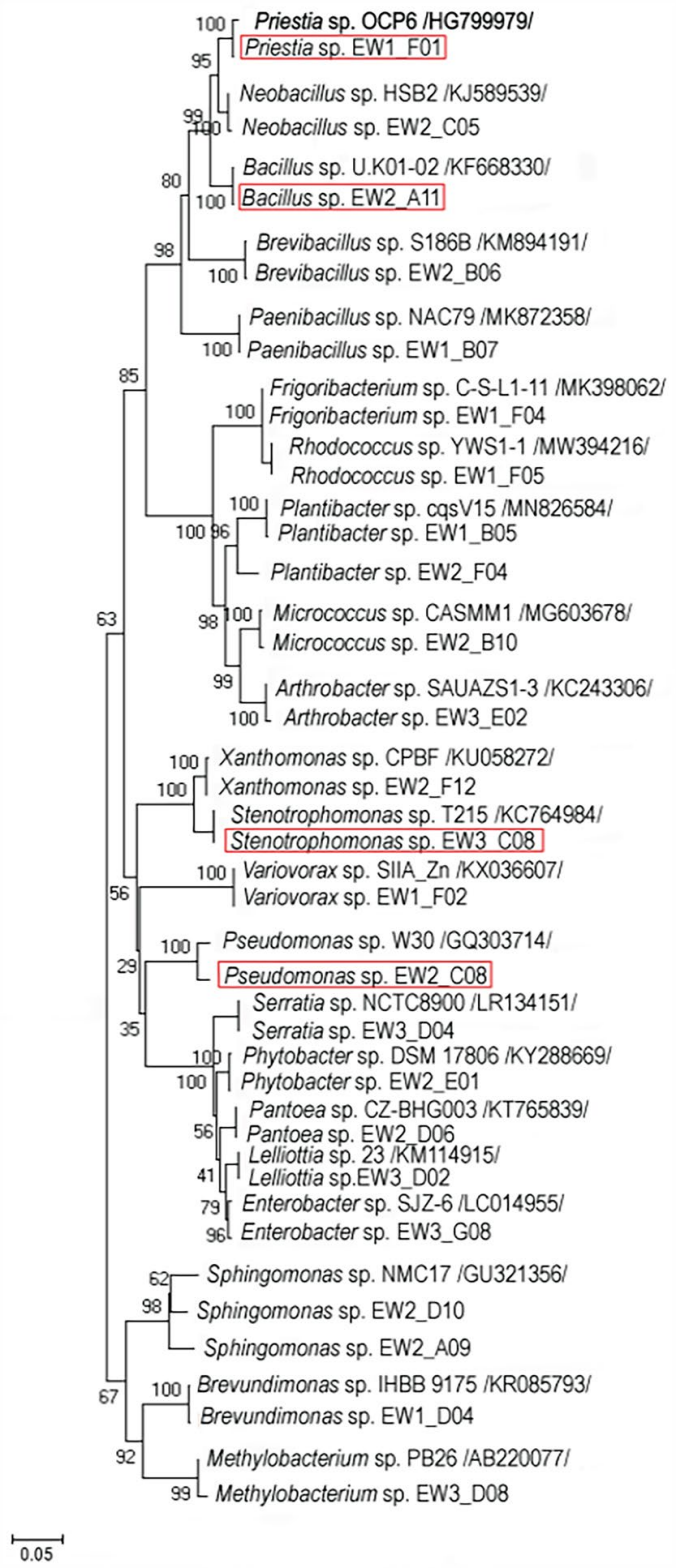
Phylogenetic Neighbor-Joining tree based on 16S rRNA gene sequences showing the relationship of studied endohytic and rhizosphere *A. arenosa* and *A. halleri* bacterial strains and reference bacteria (GenBank). Numbers at nods indicate levels of bootstap index based on analysis of 1000 resampled datasets. The scale bar indicates the number of substitutions per site. Accession numbers are shown in parentheses. Bacterial taxa proposed as potentially useful in bioremediation are marked in red rectangles.

**Figure 3:**
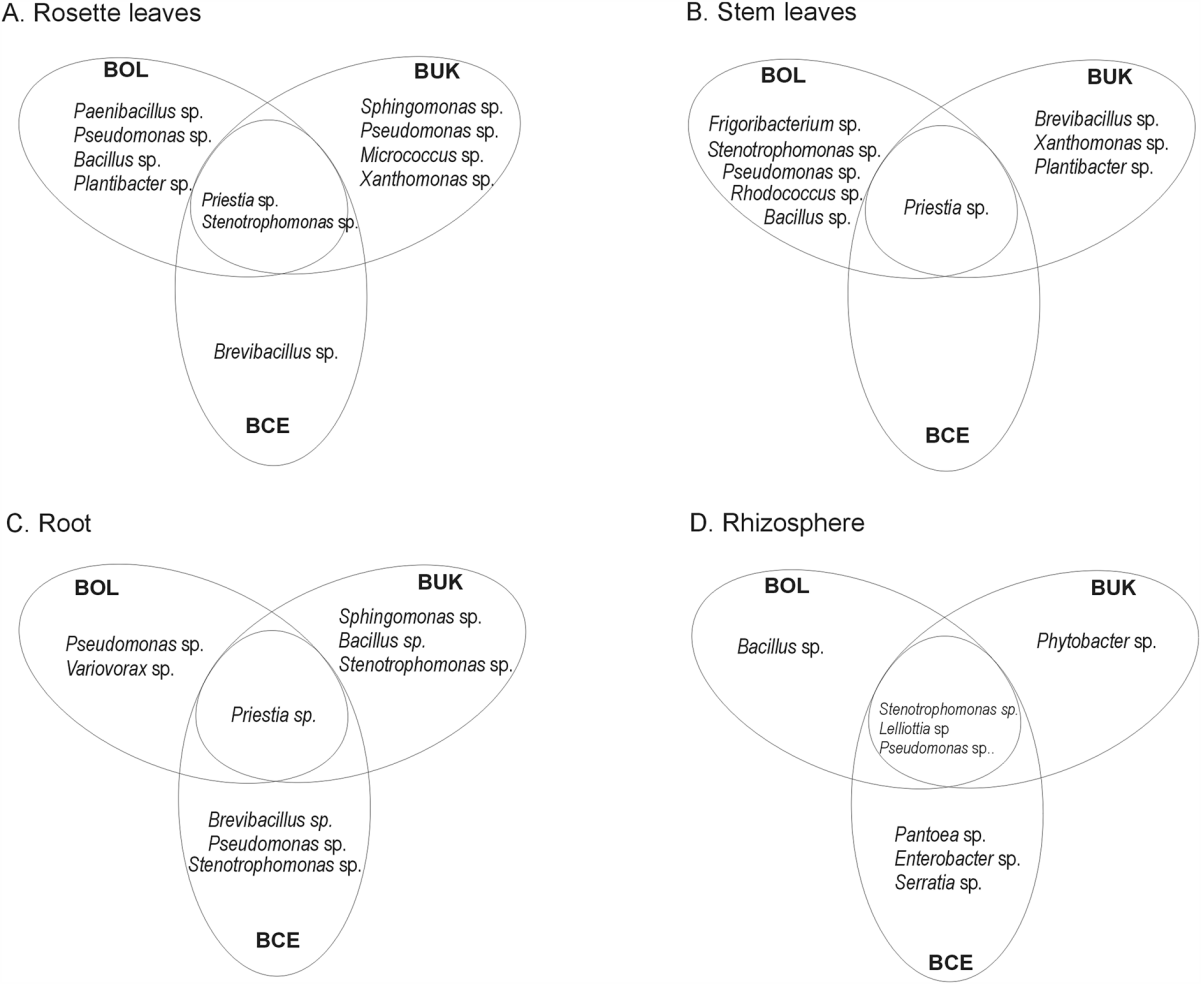
Shared and specific bacterial taxa identified as rosette leaf (**A**), stem leaf (**B**), and root (**C**) endophytes and rhizosphere (**D**) *A. arenosa* inhabitants originating from Zn–Pb–Cd Bolesław (BOL) and Bukowno (BUK) waste heaps as well as the reference Bolestraszyce (BCE) area.

The Neighbor-Joining analysis of the 16S rDNA of the cultivable bacteria showed that their taxonomic position differs between the host-plant species, type of the tissue, and origin of the plant (Fig. 2). For instance, only in rosette leaves of *A. arenosa Paenibacillus* sp. and *Micrococcus* sp. were found, whereas in stem leaves *Frigoribacterium* sp. or *Rhodococcus* sp. were identified (Fig. 3). In roots of *A. arenosa, Variovorax* sp. was found, while in its rhizosphere *Lelliottia* sp. or *Pantoea* sp. were noticed. *Xanthomonas* sp. was found only in *A. arenosa* tissues from waste heap origin. In spite of these differences, also common taxa were identified in *A. arenosa* samples from the waste heap and the reference area, i.e., *Priestia* sp. in rosette leaves, stem leaves and roots or *Stenotrophomonas* sp*.* in rosette leaves and rhizosphere.

**Figure 4:**
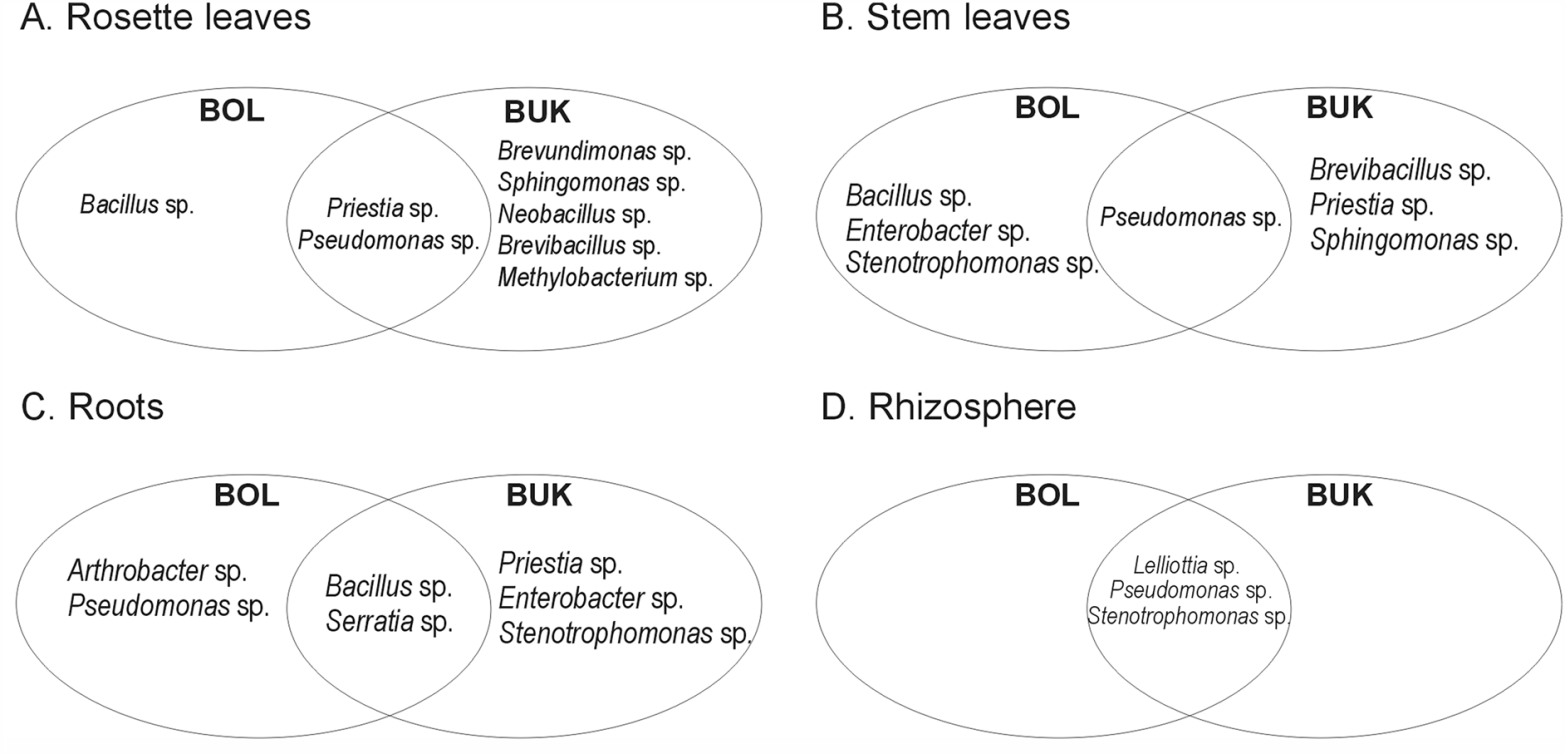
Shared and specific bacterial taxa identified as rosette leaf (**A**), stem leaf (**B**), and root (**C**) endophytes and rhizosphere (**D**) *A. halleri* inhabitants originating from Zn–Pb–Cd Bolesław (BOL) and Bukowno (BUK) waste heaps.

In the rosette leaves of *A. halleri Brevundimonas* sp., *Sphingomonas* sp., *Neobacillus* sp. or *Methylobacterium* sp. were identified, whereas in its stem leaves *Stenotrophomonas* sp. or *Pseudomonas* sp. were found (Fig. 4). In the roots of *A. halleri Arthrobacter* sp. or *Serratia* sp. were detected, while in its rhizosphere *Lelliottia* sp., *Pseudomonas* sp. or *Stenotrophomonas* sp were identified. Only in *A. halleri* tissues *Neobacillus* sp., *Methylobacterium* sp., *Arthrobacter* sp. were found. *Priestia* sp. and *Pseudomonas* sp. were noted as common genera of rosette leaf endophytes of *A. halleri* from both waste heaps. *Pseudomonas* sp. was found in the stem leaves of *A. halleri*, *Bacillus* sp. and *Serratia* sp. in the roots, while *Stenotrophomonas* sp*.*, *Pseudomonas* sp., and *Lelliottia* sp. in its rhizosphere (Fig. 4).

**Figure 5:**
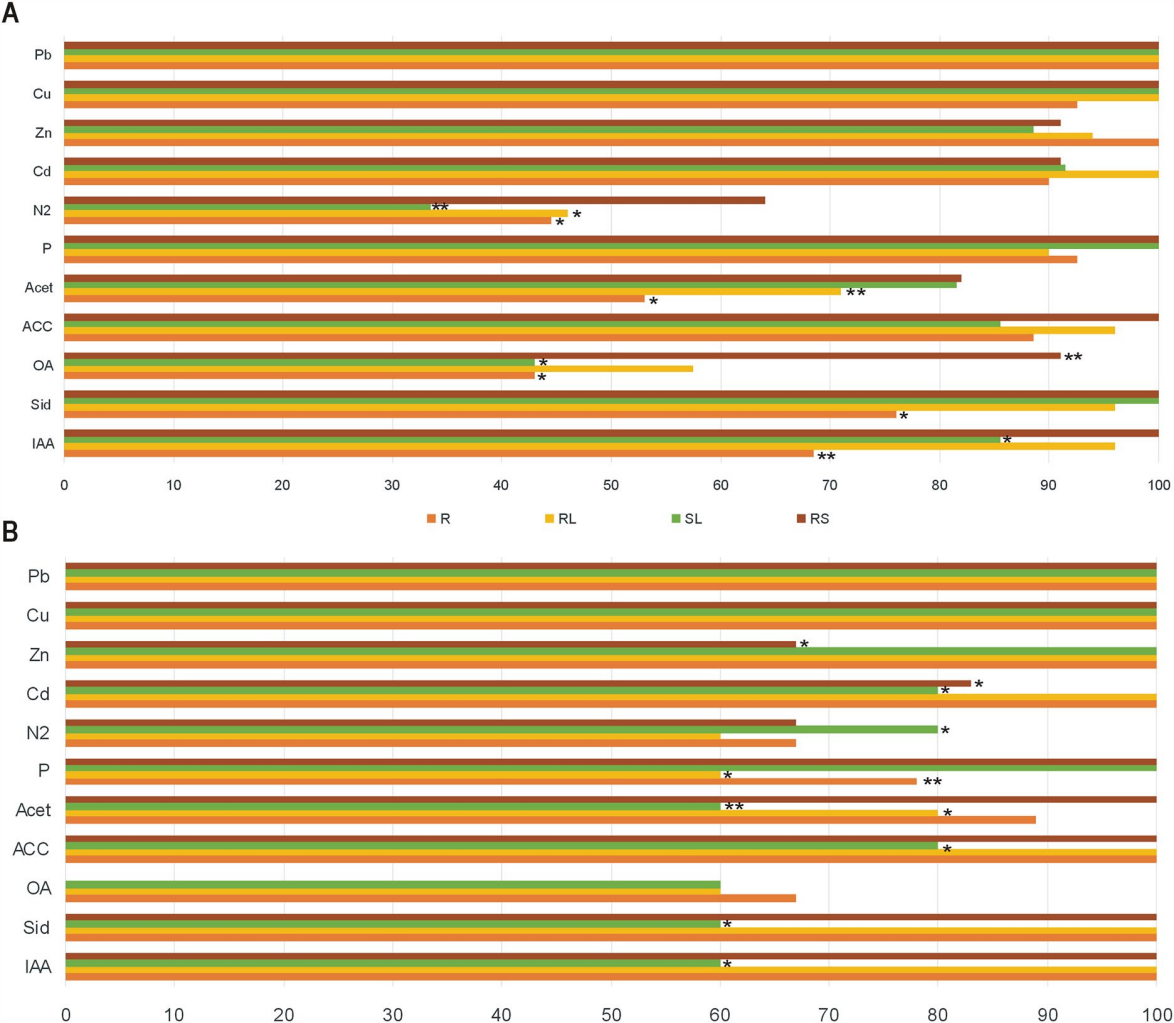
In vitro studies testing plant growth properties: indole-3-acetic acid (IAA), siderophores (Sid), organic acids (OA), and acetion (Acet) production, 1-aminocyclopropane-1-carboxylate (ACC)-deaminase activity, phosphate solubilization (P), atmospheric nitrogen fixation (N2) as well as cadmium (Cd), zinc (Zn), copper (Cu), and lead (Pb) tolerance of bacterial endophytes isolated from roots (R), rosette leaves (RL), stem leaves (SL), and rhizosphere (RS) of *A. arenosa* growing on Zn–Pb–Cd waste heaps (**A**) and the reference area (**B**).

**Figure 6:**
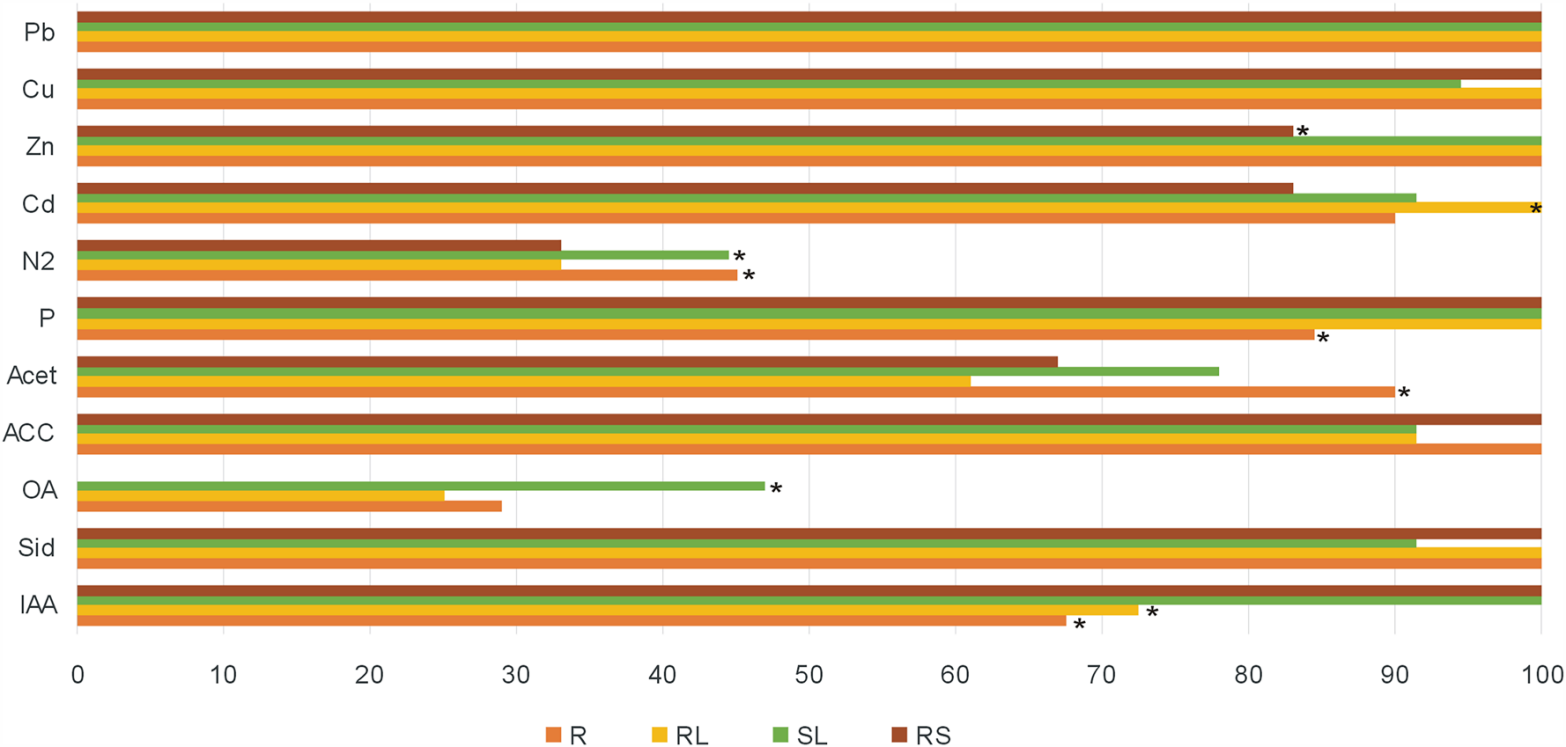
In vitro studies testing plant growth properties: indole-3-acetic acid (IAA), siderophores (Sid), organic acids (OA), and acetion (Acet) production, 1-aminocyclopropane-1-carboxylate (ACC)-deaminase activity, phosphate solubilization (P), atmospheric nitrogen fixation (N2) as well as cadmium (Cd), zinc (Zn), copper (Cu), and lead (Pb) tolerance of bacterial endophytes isolated from roots (R), rosette leaves (RL), stem leaves (SL), and rhizosphere (RS) of *A. halleri* growing on Zn–Pb–Cd waste heaps.

In vitro tests of potential plant growth promoting traits revealed differences between bacterial strains inhabiting tissues and rhizosphere of *A arenosa* (Fig. 5) and *A. halleri* (Fig. 6) depending on the host plant species, the type of the tissue as well as the origin of the plants (Table S1). Among *A. arenosa* endophytes of waste heap origin, auxins, siderophores, and acetoin could be produced by a significantly higher percentage of strains isolated from rosette and stem leaves than from roots; organic acids were produced by significantly more rosette leaf endophytes than stem leaf and root endophytes. Phosphate solubilization was performed by all isolated stem leaf endophytes while a significantly higher percentage of rosette leaf and root endophytes than stem leaf endophytes could perform atmospheric nitrogen fixation (Fig. 5A). 100% of the rhizosphere bacteria from *A. arenosa* of the waste heap origin showed able to synthesize auxins, siderophores, ACC, and to solubilize phosphates, 92% produced organic acids, 83% of the strains synthesized acetoin, and 64% could fix atmospheric nitrogen (Fig. 5A). In *A. arenosa* originating from the reference area, significantly more (100%) endophytic strains from rosette leaves and roots and also from the rhizosphere could synthesize auxins and siderophores in comparison with the strains from the stem leaves (60%) (Fig. 5B). About 60% of the endophytes from rosette and stem leaves as well as from the roots of *A. arenosa* from the Bolestraszyce reference area synthesized organic acids, but rhizosphere bacteria did not produce them. Bacteria associated with *A. arenosa* of reference area origin showed efficient in producing ACC deaminase, phosphate solubilization and atmospheric nitrogen fixation; all strains isolated from roots, rosette leaves and rhizosphere were effective in ACC deamination; 100% of the stem leaf endophytes and rhizosphere inhabitants could solubilize phosphate; 80% of the stem leaf endophytes could fix atmospheric nitrogen (Fig. 5B). No significant differences were observed in Cd, Zn, Cu, and Pb tolerance of bacteria associated with *A. arenosa* originating from the waste heap area, but significantly lower tolerances to Zn and Cd were found in rhizosphere strains isolated from *A. arenosa* growing on the reference area (Fig. 5, Table S1).

All endophytes isolated from the stem leaves and rhizosphere of *A. halleri* could produce indole-3-acetic acid; siderophores were produced by all studied root, rosette leaves, and rhizosphere bacteria; ACC deaminase was produced by all isolated root endophytes and rhizosphere bacteria; organic acids were synthesized by 46% of the stem leaf endophytes and were not produced by all rhizosphere bacteria; acetoin was produced by 90% of the root endophytes; the highest frequency of phosphorus solubilization was found for root and stem leaf endophytes and rhizosphere bacteria; the highest percentage of atmospheric nitrogen fixation (45%) was found for the root endophytes (Fig. 6).

A significantly higher percentage of *A. arenosa* rosette leaf endophytes synthesized IAA, organic acids, acetoin, and fixed atmospheric nitrogen in comparison with those of the *A. halleri* rosette. Significantly more *A. halleri* stem leaf endophytes produced auxins compared to *A. arenosa* (Figs. 5 and 6). All *A. halleri* endophytes and rhizosphere inhabitants were tolerant to Pb and Cu. A higher percentage of *A. halleri* endophytes were tolerant to Zn in comparison to the rhizosphere inhabitants; more rosette leaves endophytes were tolerant to Cd in comparison to strains isolated from other tissues (Fig. 6).

**Figure 7:**
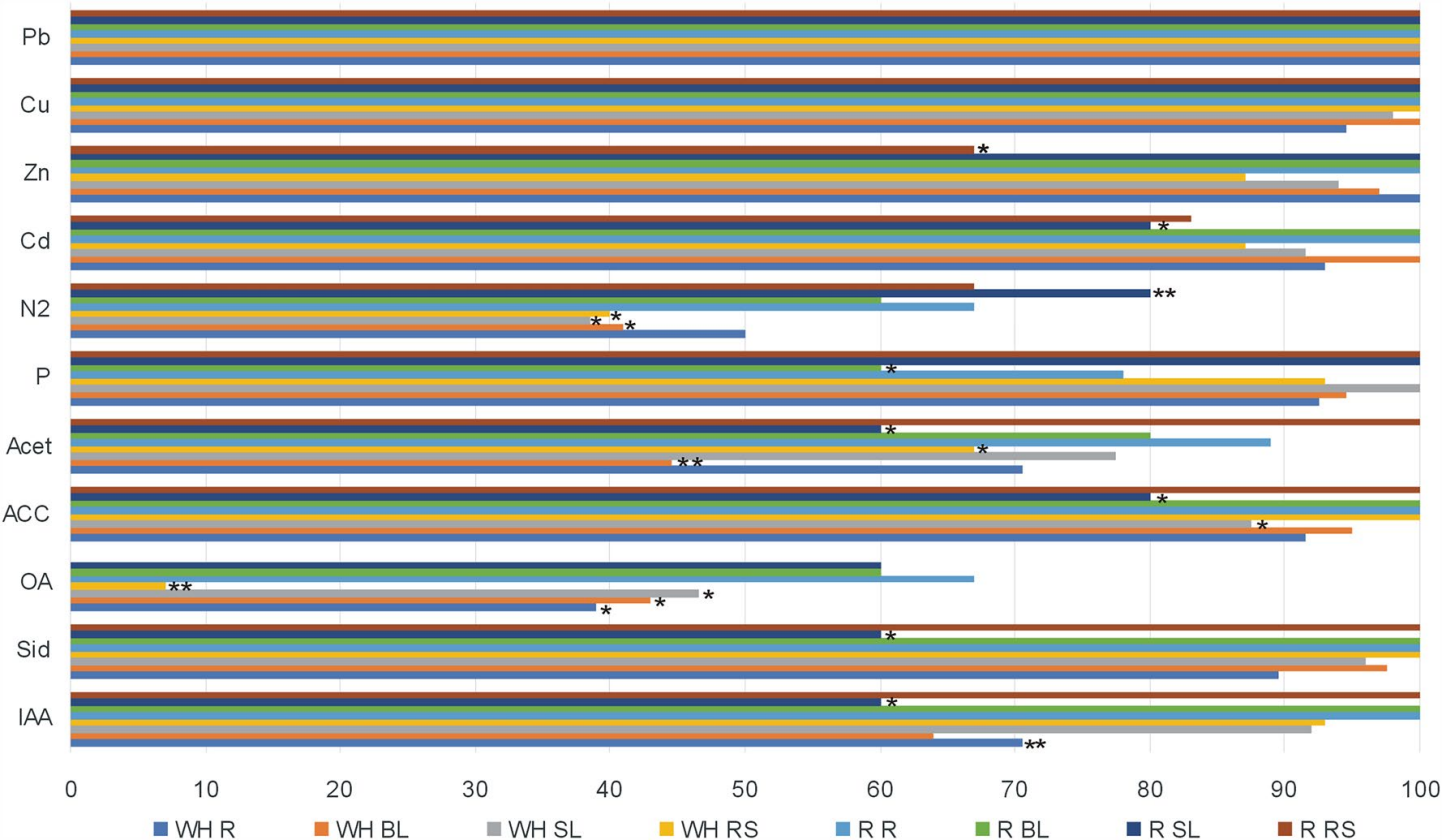
Comparison of in vitro studied plant growth properties: indole-3-acetic acid (IAA), siderophores (Sid), organic acids (OA), and acetion (Acet) production, 1-aminocyclopropane-1-carboxylate (ACC)-deaminase activity, phosphate solubilization (P), atmospheric nitrogen fixation (N2) as well as cadmium (Cd), zinc (Zn), copper (Cu), and lead (Pb) tolerance of bacteria isolated from roots (R), rosette leaves (RL), stem leaves (SL), and rhizosphere (RS) of hyperaccumulator plants *A. arenosa* and *A. halleri* originating from Zn–Pb–Cd waste heaps (WH) and reference (R) area.

The results from in vitro studies revealed differences in plant growth promotion traits between the bacterial endophytes of the hyperaccumulators originating from the waste heap and the reference area. Significantly more endophytes of roots, stem and rosette leaves and rhizosphere from the reference area could fixate atmospheric nitrogen compared to the ones from waste heap origin. Significantly more stem leaf endophytes of the waste heap origin could produce auxins, siderophores, and acetoin than the stem leaf endophytes of plants from the reference grassland; more rosette leaf endophytes of waste heap origin could solubilize phosphate in comparison with those originating from the reference area (Fig. 7). Bacterial communities associated with *A. arenosa* and *A. halleri* growing on the Bolesław and Bukowno waste heaps and the reference area did not show significant differences concerning IAA production, ACCD activity and SI (Table S2).

Among the bacterial strains from the waste heap, which were tolerant to Zn, Pb, and Cd and showed positive for all plant growth promotion traits that were tested in vitro, nine endophytic strains, six from *A. arenosa* origin as well three from *A. halleri*, might have significant potential for use in bioremediation (Table S1). The most promising endophytes of *A. arenosa* from the Bolesław waste heap area were: *Priestia* sp. strain EW1_D06 isolated from rosette leaves, *Bacillus* sp. strain EW1_B04, *Pseudomonas* sp. strain EW1_E01, and *Stenotrophomonas* sp. strain EW3_G06 that inhabited stem leaves. From the Bukowno waste heap it were: *Priestia* sp*.* strain EW1_H08 isolated from rosette leaves as well as *Pseudomonas* sp. strain 1.2 of rhizosphere origin. From the *A. halleri* endophytes the most promising were: *Bacillus* sp. strain EW2_B02 isolated from rosette leaves (Bolesław origin), *Bacillus* sp. strain EW2_H12 isolated from stem leaves (Bolesław origin), and *Priestia* sp*.* strain EW1_C08 isolated from roots (Bukowno origin).

Trace element analysis revealed significantly higher Zn, Pb, and Cd concentrations in rhizosphere soil as well as in rosette and stem leaves and roots of *A. arenosa* growing on the Bolesław and Bukowno waste heaps compared to those from the Bolestraszyce reference area (Table 2B). Zinc concentrations in leaves and roots of *A. arenosa* and *A. halleri* were significantly higher level than those of Pb and Cd. In leaves of plants of the Bolesław origin Zn concentrations were higher than in roots. The Pb concentrations in roots of *A. arenosa* and *A. halleri* were significantly higher than in the leaves (Table 2). The limits of metal detection (LOD) as well as limits of metal quantifications (LOQ) were detected as follows: LOD_Zn_ = 0.040 and LOQ_Zn_ = 0.13 μg × L^−1^, LOD_Pb_ = 0.0031 and LOQ_Pb_ = 0.0102 μg × L^−1^, LOD_Cd_ = 0.0017 and LOQ_Cd_ = 0.0056 μg × L^−1^.

**Table 2:** Zinc (Zn), lead (Pb), and cadmium (Cd) mean concentrations in roots (R), rosette leaves (RL), stem leaves (SL), and rhizosphere (RS) of *A. halleri* (A) and *A. arenosa* (B) growing on the Bolesław (BOL) and Bukowno (BUK) waste heap areas as well as the reference Bolestraszyce (BCE) one.

	*A. halleri* BOL [mg kg^−1^]				*A. halleri* BUK [mg kg^−1^]							
	RS	RL	SL	R	RS	RL	SL	R				
(A.)												
Zn	498 ± 27.73	11,656 ± 1,768^a^	5027 ± 1,314^b^	945 ± 581	6075 ± 1,486^a^	1264 ± 282^b^	4134 ± 64.63^a^	4233 ± 886^a^				
Pb	82.72 ± 37.47	26.26 ± 6.45	20 ± 8	314 ± 148^a^	1698 ± 485	12.08 ± 0.96^a^	182 ± 70.89^b^	378 ± 129^b^				
Cd	5.51 ± 1.37	0 ± 0^a^	0 ± 0^a^	3.29 ± 0.99	31.59 ± 8.61	3.46 ± 4.89^a^	1.98 ± 0.79^a^	26.29 ± 6.72				

	*A. arenosa* BOL [mg kg^−1^]				*A. arenosa* BUK [mg kg^−1^]				*A. arenosa* BCE [mg kg^−1^]			
	RS	RL	SL	R	RS	RL	SL	R	RS	RL	SL	R
(B.)												
Zn	502 ± 31.49	2445 ± 238^a^	3522 ± 136^b^	761 ± 128^c^	7856 ± 1320	2782 ± 172^a^	5136 ± 284^b^	3160 ± 971^a^	83.41 ± 2.52	108 ± 48	231 ± 71	119 ± 51
Pb	79.87 ± 12.69	13 ± 5^a^	0 ± 0^b^	288 ± 129^c^	2418 ± 526	106 ± 48^a^	71 ± 10^a^	1115 ± 199^b^	9.86 ± 2.20^a^	2.89 ± 2.19	1.90 ± 0.40	3.32 ± 0.25
Cd	4.89 ± 0.97	0 ± 0^a^	0 ± 0^b^	13.12 ± 1.78^c^	32 ± 6.61	13 ± 5^a^	1.32 ± 0.26^b^	15 ± 2^a^	2.51 ± 0.12^a^	0 ± 0	1.13 ± 0.02	0 ± 0

Values are represented as means ± SD (n = 5). Significant differences between groups were marked with different letters.

## Discussion

Our study of 144 cultivable bacterial endophytes isolated from roots, rosette and stem leaves as well as rhizosphere of the Zn-hyperaccumulators *A. halleri* and *A. arenosa* growing on calamine waste heaps in Bolesław and Bukowno and the unpolluted reference area in Bolestraszyce revealed different percentages of intra-community taxonomic diversity of bacteria, high tolerance to Zn, Pb, Cd, and Cu as well as a prominent potential to beneficially influence the fitness and growth of the host plants (Figs. 1, 2, 5, 6 and Table S1). Almost all *A. arenosa* and *A. halleri* rhizosphere bacterial communities, except *A. arenosa* rhizosphere bacteria of Bukowno waste heap origin, showed maximal 100% strain diversity. The highest level of the Shannon’s diversity index (*H’* = 2.01) was found for the *A. halleri* rosette leaf endophyte community originating from the Bukowno waste heap while the lowest one (*H’* = 0) was observed for the *A. arenosa* stem leaf endophytes from the reference area origin (Table 1).

Bacteria can develop different tolerance mechanisms to cope with metal toxicity, e.g. by modifications of cellular barriers, efflux of metals out of the cell, enzymatic conversion of metals, extracellular and intracellular sequestration^29,31,32,53^. Bacteria can positively stimulate plant growth via increasing the availability of essential nutrients, production and regulation of compounds regulating plant growth or protecting plants against diseases^33–35^. Among the bacteria found in the present study, several have been mentioned for possessing bioremediation potential, i.e., *Arthrobacter* sp., *Bacillus* sp., *Brevundimonas* sp., *Enterobacter* sp., *Frigoribacterium* sp., *Lelliottia* sp., *Micrococcus* sp., *Methylobacterium* sp., *Neobacillus* sp., *Paenibacillus* sp., *Pantoea* sp., *Phytobacter* sp., *Plantibacter* sp., *Pseudomonas* sp., *Rhodococcus* sp., *Serratia* sp., *Sphingomonas* sp., *Stenotrophomonas* sp., *Variovorax* sp., *Xanthomonas* sp.^54–59^. The adaptations of bacteria to metal toxicity are determined genetically on chromosomal genes and/or genes located on plasmids^60,61^. In general, the genetics of metal tolerance mechanisms in bacteria are quite well-studied. For example, several species of genus *Bacillus* comprise strains that are efficient in removal of Zn (e.g.,* B. subtilis*, *B. licheniformis*, *B. cereus*, *B. jeotgali*, *B. firmus*), Cd (e.g.,* B. subtils*, *B. licheniformis*, *B. safensis*, *B. megaterium*, *B. catenulatus*) or Pb (e.g.,* B. cereus*)^54,62^. Cadmium resistance of *Bacillus* sp. is determined by the *cad* operon (3.5 kbp) localized on plasmid pI258, composed of *cadA* and *cadC* genes. Gene *cadA* encodes a 727-aminoacid protein that functions as Cd efflux ATP-ase, while *cadC* encodes a 122-aminoacid protein, which acts as a regulator of the *cad* operon^63^. In contrast, Pb^2+^ ions in *Bacillus* spp. cells are immobilized by phosphate, carboxyl, carbonyl, sulfhydryl, and hydroxyl groups that give cell walls a negative charge as well as in polypeptides, polysaccharides, and proteins via van der Waal’s forces, covalent and ion bonds. The resistance to lead in *Bacillus* sp. is determined by a plasmid *pbr* operon^54,61^.

Metal tolerant bacteria that can immobilize metal ions can have indirect beneficial effects on the growth of their host plants^33,64,65^. In vitro studies on plant growth promotion traits of *A. halleri* and *A. arenosa* endophytes and rhizosphere inhabitants showed that bacterial traits differ between plant tissues, plant species and their origin (Figs. 5, 6, 7 and Table S1). A significantly higher percentage of *A. arenosa* root endophytes originating from the waste heap area could solubilize phosphate compared to those originating from the reference area. Similarly, higher percentages of endophytes of basal leaves of plants from the waste heaps were producing acetoin and solubilizing phosphate compared to the ones from the reference grassland. Also higher percentages of stem leaf endophytes from waste heap origin could synthesize of auxins, siderophores, and acetoin in comparison to endophytes of *A. arenosa* growing on the reference grassland (Fig. 5). Metabolic traits were also different between host plant species. Higher percentages of root endophytes of *A. halleri* could produce siderophores, ACC-deaminase, and acetoin than root endophytes of *A. arenosa* of waste heap origin. Similarly, a higher percentage of *A. halleri* basal leaf endophytes could solubilize phosphates, as well as more *A. halleri* stem leaf endophytes could produce auxins and ACC-deaminase in comparison to endophytes of *A. arenosa* from the waste heaps (Figs. 5 and 6). 1-aminocyclopropane-1-carboxylate (ACC) deaminase (ACCD) converts the immediate precursor of ethylene in plant cells (ACC) into α-ketobutyrate and ammonia^33,66,67^, resulting in lowered levels of ethylene production. High levels of ethylene can negatively affect plant growth e.g. by inhibiting root and stem elongation, by inducing hypertrophy and accelerating senescence and abscission^68^. It is also known that, in case of metal stress, phosphate solubilizing bacteria (PSB), e.g.* Bacillus* spp., *Enterobacter* spp., *Micrococcus* spp., *Pseudomonas* spp., *Rhizobium* spp.^69^, are of the great importance for plant growth by enhancing the availability of phosphorous, which is an essential building block of several macromolecules e.g., DNA, RNA, ATP and phospholipids^70–72^. *Stenotrophomonas* spp. strain RC5, *Serratia* spp. strain RCJ6 and *Enterobacter* spp. strain RJAL6 that could solubilize phosphate by producing organic acids and phosphatases were demonstrated to assist ryegrass to overcome aluminium toxicity^73^, while *Ensifer adhaerens* strain OS3 with high acid and alkaline phosphatase activity turned out to be a chromium reducer^74^. Under metal stress conditions also bacteria synthesizing siderophores, low molecular (400–1500 Da) chelators of iron (FeIII)^75^, have positive effects on plant growth and health. Iron is essential for maintaining the structure and function of chloroplasts, DNA synthesis, cellular respiration, and is involved in many redox reactions, among others as a constituent of many enzymes’ prosthetic groups^76^. Presence of metals induces the synthesis of siderophores in bacteria. For example, under Cd(II) and Zn(II) stress *Pseudomonas aeruginosa* strain ZGKD3 produced pyoverdine^77^, under Cd (II) stress *Streptomyces* spp. isolated from *Betula pendula* and *Alnus glutinosa* rhizosphere produced hydroxamate, catecholate and phenolate siderophores, particularly ferrioxamine B^78^, while under exposure to toxic Pb(II) concentrations *Bacillus* spp. strain PZ-1 isolated from *Brassica juncea* synthesized siderophores of hydroxamate structure^79,80^. Bacteria associated with plants can synthesize phytohormones, e.g. auxins, and/or influence the hormone balance of their host plants, e.g. by, like already mentioned above, lowering the plants’ ethylene production by ACCD activity^81^. Under conditions of metal stress, the IAA-synthesizing *B. megaterium* strain MCR-8 enhanced the biomass production of *Vinca rosea* as well as increased the levels of phenols, flavonoids, and antioxidative enzymes^82^, while IAA-producing *Leifsonia* spp. and *Bacillus* spp. significantly increased the growth of *Zea mays* in comparison to non-polluted soils^83^. Acetoin (3-hydroxy-2- butanone), one of the volatile organic compounds (VOCs), that are low molecular weight (< 300 Da) hydrocarbons emitted in a gaseous phase or secreted into liquids^84,85^, provokes ISR (induced systemic resistance) and contributes to an improvement of plant growth^86,87^. Bacterial born VOCs influence the bacterial motility, antibiotic resistance or biofilm formation, as well as influence the plants, e.g. increase biomass, fruit yield, seed production, lateral root and root hair formation, nutrient uptake, and photosynthetic activity^87–90^. It is clear that there are some strains of bacteria active in deaminase ACC, phosphate solubilization as well as in production of organic acids, siderophores, auxins, and VOCs under metal stress conditions, and are beneficial to plants factors of the potential usage in restoration of degraded soils.

In the present research we identified strains with high tolerance to Zn, Pb, Cd that also tested positive for all in vitro potential plant growth promotion parameters. Therefore, we propose them as potential candidates for bioremediation purposes. Among these strains of the waste heap origin, six strains associated with *A. arenosa* and three *A. halleri* endophytes meet these criteria. From *A. arenosa* originating from the Bolesław waste heap it are *Priestia* sp. strain EW1_D06 (isolated from rosette leaves), *Bacillus* sp. strain EW1_B04, *Pseudomonas* sp. strain EW1_E01, and *Stenotrophomonas* sp. strain EW3_G06 (from the stem leaves), and from the Bukowno waste heap origin it are *Priestia* sp. strain EW1_H08 (from basal leaves) as well as *Pseudomonas* sp*.* strain 1.2 (from the rhizosphere). From *A. halleri* orginating from Bolesław it are *Bacillus* sp. strain EW2_B02 (isolated from rosette leaves) and *Bacillus* sp. strain EW2_H12 (from stem leaves) and from Bukowno *Priestia* sp. strain EW1_C08 (from roots). *Priestia* sp. (formerly *Bacillus* sp.) was shown to be efficient to assist their host plant to remove Pb and Cd from polluted soil^58,91,92^ and equipped with plant growth promoting traits^93^. For example, *P. megaterium* strain R181 was reported as an effective stimulator of corn and wheat growth^94^ and also as an inhibitor of plant diseases due to synthesis of antibiotic-type compounds, e.g. lipopeptides similar to surfactins, lichenysins, itrurinA, and fengycins^95,96^. Also, biosorptive microbes like *Stenotrophomonas maltophilia* as well as *Pseudomonas viridiflava*^97^ were suggested as useful in metal bioremediation^98,99^. It was also reported that *S. maltophilia* producing ACC deaminase, gibberellic acid, indole-3-acetic acid, and siderophores can promote host plant growth^100^.

In conclusion, our studies revealed that *A. arenosa* and *A. halleri* are Zn-hyperaccumulators, which store high metal concentrations in leaves and in roots. Endophytic and rhizospheric bacterial communities associated with these hyperaccumulators differ in taxonomic composition and also in metabolic traits depending on the plant species and origin of the host-plant and the plant compartment. Due to a high tolerance to Zn, Pb, and Cd and the fact that they tested positive for all in vitro assessed plant growth promotion traits, bacterial strains originating from the waste heaps, i.e., *Priestia* sp*.*, *Stenotrophomonas* sp*.*, *Pseudomonas* sp*.*, and *Bacillus* sp. are proposed as potential candidates for bioremediation purposes.

## Materials and methods

In total 144 bacterial strains were isolated from plant tissues (123 strains), i.e., roots, rosette (basal) leaves, and stem leaves as well as from the rhizosphere (21 strains) of *A. halleri* and *A. arenosa* from two, about 100-yrs old Zn–Pb–Cd polluted waste heaps in Bolesław (50° 17′ N 19° 29′ E) and Bukowno (50° 16′ N 19° 28′ E) situated in the Olkusz Ore Region (Silesia-Krakow Upland), and from a reference grassland in Bolestraszyce (49° 48′ N 22° 50′ E, Przemyskie Foothills), in Southern Poland. The plants were randomly selected and dug out with a sterilized shovel, stored in sterile plastic bags that were kept and transferred to the laboratory in a temperature controlled cool box (4–8 °C). Collected *A. halleri* and *A. arenosa* individuals were identified by Dr. Ewa Oleńska and the voucher specimens were deposited in Faculty of Biology University of Bialystok (Poland) plant collection.

### Isolation of plant associated bacteria

#### Rhizosphere bacteria

Approximately 5 g of soil was suspended in 30 mL liquid R2A medium (in w/v%, casein acid hydrolysate 0.05, yeast extract 0.05, protease peptone 0.05, dextrose 0.05, soluble starch 0.05, K_2_HPO_4_ 0.03, MgSO_4_ × 7H_2_O 0.003, sodium pyruvate 0.03, agar 1.5, pH 7.2)^101^ in sterile Falcon tubes and incubated on a rotary shaker (140 rpm) at 28 °C. After seven days tubes were centrifuged at 2000 rpm for 15 min and 100 µL of supernatant diluted into 10^−8^ was plated on Petri dishes with solid R2A medium. Single bacterial colonies of a different morphology were transferred to plates with solid R2A medium. After 7-days of incubation at 28 °C in a liquid R2A medium they were transferred into sterile deionized water containing glycerol (15% w/v) and 0.85% w/v NaCl, and stored in a freezer at − 45 °C.

#### Plant tissue endophytes

Basal and stem leaves as well as roots were carefully rinsed with water and then surface sterilized with 0.1% NaOCl (6–14% active chlorine, Emplura^®^) for 10 s. Subsequently, the tissues were transferred into 75% ethanol for 30 s, and rinsed three times in sterile MilliQ water for 30 s. The effectiveness of sterilization was verified by plating 100 µL of the last rinsing water on a solid 1/10 869 medium (in w/v%, CaCl_2_ × 2H_2_O 0.035, D-glucose 0.1, NaCl 0.5, tryptone 1.0, yeast extract 0.5, agar 1.4, pH 7.0), which is an appropriate medium for studying the diversity of plant endophytes^102^. Leaves and roots of plants were homogenized in sterile 10 mM MgSO_4_ buffer solution using TissueLyser LT (Qiagen); thereafter, 100 µL of plant extract diluted into 10^−4^ was plated on 1/10 869 solid medium. After 7 days of incubation at 30 °C, single colonies that showed different morphologies were plated on separate 1/10 869 solid media. Pure colonies were transferred to sterile liquid glycerol medium, and stored in a freezer at − 45 °C.

The level of the intra-population taxonomic diversity was estimated using the index of strain diversity (*ISD*) that expresses population richness in bacterial strains and the Shannon’s diversity index (*H*’), which indicates a population bacterial strain variability. Parameters *ISD* and *H*’ were calculated according to equations described by Oleńska and Małek^103^.

### Identification of bacteria using 16S rRNA gene analysis

DNA was isolated from each of the 144 bacterial strains using the Applied Biosystems MagMAX™ Total DNA Multi-Sample Ultra Kit according the manufacturer’s instructions (ThermoFisher Scientific) after lysis of the bacterial cells as described by Oleńska et al.^36^. The amplification of the 16S rRNA gene was performed using FastStart™ HF (High Fidelity) PCR System (Sigma-Aldrich) in a total volume 25 μL containing: 2.55 µL 10 × concentrated with 18 mM MgCl_2_ FastStart HF Reactive Buffer, 0.5 µL 10 mM PCR grade dNTP (deoxynucleoside triphosphate) mix, 0.5 µL 0.2 µM of each primer, 0.2 µL 5 U/µL Fast Start High Fidelity Enzyme Blend, 19.75 µL nuclease free water, and 1 μL of DNA as a template. Amplification of 16S rDNA was performed with the primers 27F 5’-AGAGTTTGATCMTGGCTCAG-3’ and 1492R 5’-TACGGYTACCTTGTTACGACTT-3’ (Macrogen, Netherlands)^104^ using the following conditions: initial denaturation at 94 °C for 5 min, 35 cycles of denaturation at 94 °C for 1 min, annealing at 52 °C for 30 s, and extension at 72 °C for 3 min, and a final extension at 72 °C for 10 min. Amplicons were inspected in a 1% agarose gel electrophoresis performed in 1 × TBE buffer, and documented with the Gel Doc System (Invitrogen). The Sanger sequence PCR reaction and removal of fluorescent labelled ddNTPs unbound with DNA were performed by Macrogen (Netherlands). The 16S rRNA gene sequences obtained in the present study were inspected using the Chromas 2.5 and BioEdit^105^ programs. The high-quality sequences were searched for the closest relative in the NCBI (National Center for Biotechnology Information) database using BLAST (Basic Local Alignment Search Tool).

### In vitro analysis of plant growth promotion traits

Each bacterial strain was in vitro tested for traits that can potentially improve plant growth by: (1) increasing the availability of nutrients, e.g. phosphate solubilization, synthesis of siderophores and organic acids, and atmospheric nitrogen fixation; (2) synthesis of potential plant growth regulators i.e., indole-3-acetic acid and 1-aminocyclopropane-1-carboxylate (ACC)-deaminase (ACCD) and acetoin production. Most in vitro phenotypic tests were qualitatively, colorimetrically assessed. A phosphate solubilization index (SI) was estimated by plating bacteria on selective NBRIP medium; IAA production as well as ACCD activity were quantitatively, spectrophotometrically assessed. The ability to synthesize organic acids was studied according to Cunnigham and Kuiack^106^, siderophore production was tested using chrome-azurol S according to Schwyn and Neilands^107^ using a 284 medium^108^. Bacterial acetoin production was examined according to Romick and Fleming^109^. The bacterial capacity to solubilize phosphate was tested according to Pikovskaya^110^ with the modifications of Nautiyal^111^, and the phosphate solubilization index (SI) was calculated using an equation described by Pande et al.^112^. The capability of bacteria to synthesize IAA was investigated according to the methods described by Gordon and Weber^113^ and Patten and Glick^114^. Quantification of the synthesized IAA by bacteria was performed according to Penrose and Glick^115^ and the bacterial ACCD activity was studied according to Belimov et al.^116^ and was described in detail in Oleńska et al.^36^.

### Metal concentrations in plant tissues and soil

Zn, Pb, and Cd concentrations were examined in five samples of rhizosphere soil as well as roots, basal and stem leaves collected from *A. arenosa* and *A. halleri* growing on the Bolesław and Bukowno Zn–Pb–Cd waste heaps and on the reference Bolestraszyce area. The mineralization of plant tissues and soil samples was performed in a Mars 6 microwave oven (CEM Corporation, Matthews, NC, USA) according to the procedure described by Oleńska et al.^36^. The quality assurance procedures involving analysis of reagent blanks and reference materials (Montana II soil 2711a NIST^®^ SRM^®^ and Tomato leaves 1573a NIST^®^ SRM^®^, Sigma-Aldrich) were performed in parallel. Metal concentrations were determined using ICP-MS 2030, Shimadzu, Japan equipped in mini-torch (quartz), 1.2 kW radio frequency power generator, under argon gas flow. Zn, Pb, and Cd concentrations were expressed as μg × L^−1^, and the quantified isotopes were used as follows Zn^66^, Pb^208^, and Cd^111^.

### Statistical analysis

Metal as well as IAA concentrations, ACCD activity, and SI index results were presented as means ± SD. Values were analyzed with one-way analysis of variance (ANOVA), and significant differences between means were estimated with the multiple range Duncan’s test using Statistica version 13 (TIBCO). Differences in *H’* and *ISD* values between populations were determined with the usage of non-parametric U Mann–Whitney statistical test at the significance level *p* < 0.05. The 16S rRNA gene based phylogenetic analysis of the bacterial taxa was performed using MEGA 7.0 software^117^. The phylogenetic Neighbor Joining tree construction was based on an analysis of 1000 resampled data sets according to the Maximum Composite Likelihood model.

### Permissions or licences and legislation statement

The collection of, i.e.,* Arabidopsis halleri* and *Arabidopsis arenosa*, being the source material of bacteria analysed in present study, does not need to obtain any permissions or licenses in Poland. Both species are not under species protection and were not sampled from protected areas.

I confirm that the experimental research and field studies presented in actual manuscript, including the collection of plant material and all used methods were carried out in accordance with institutional, national, and international guidelines and legislation.

### Supplementary Information

Supplementary Tables.

## Data Availability

The datasets analysed during the current study are available in Supplementary Material of this manuscript. The 16S rRNA gene sequences were deposited in NCBI GenBank database under accession numbers OQ151829-OQ151972.
