# Supplementary material for: Bacteria associated with Zn-hyperaccumulators Arabidopsis halleri and Arabidopsis arenosa from Zn–Pb–Cd waste heaps in Poland as promising tools for bioremediation
Source: Sci Rep. 2023 Aug 3;13:12606. doi: 10.1038/s41598-023-39852-6 (PMC10400580; doi:10.1038/s41598-023-39852-6)
Supplement: Supplementary file 1 — Supplementary Tables. [file 41598_2023_39852_MOESM1_ESM.docx]

Supplementary Material

Table S1. Rhizospheric (RS) and endophytic bacterial strains isolated from roots (R), rosette leaves (RL), and stem leaves (SL) of hyperaccumulators *A. halleri* (H) and *A. arenosa* (A) growing on Zn-Pb-Cd Bolesław (Bol) and Bukowno (Buk) waste heaps as well as the reference Bolestraszyce (Bce) area and their metabolic traits, i.e., IAA - indole-3-acetic acid, Sid – siderophores, OA – organic acids, ACC – 1-aminocyclopropane-1-carboxylate, Acet – acetoin, P – phosphate solubilization, N_2_ – atmospheric nitrogen fixation, tolerance to cadmium (Cd), zinc (Zn), copper (Cu), and lead (Pb).

| No. | Bacterial name | strain | GenBank no. | Strain origin | IAA | Sid | OA | ACC | Acet | P | N_2_ | Cd | Zn | Cu | Pb |
| --- | --- | --- | --- | --- | --- | --- | --- | --- | --- | --- | --- | --- | --- | --- | --- |
| 1 | *Priestia* sp. | EW1_F01 | OQ151829 | A, Bol, R | 1 | 1 | 0 | 0 | 1 | 1 | 1 | 1 | 1 | 1 | 1 |
| 2 | *Variovorax* sp. | EW1_G01 | OQ151830 | A, Bol, R | 1 | 1 | 1 | 1 | 0 | 1 | 1 | 1 | 1 | 1 | 1 |
| 3 | *Variovorax* sp. | EW1_F02 | OQ151831 | A, Bol, R | 1 | 1 | 0 | 1 | 0 | 1 | 1 | 1 | 1 | 1 | 1 |
| 4 | *Priestia* sp. | EW1_A03 | OQ151832 | A, Bol, R | 1 | 1 | 1 | 1 | 0 | 0 | 1 | 1 | 1 | 1 | 1 |
| 5 | *Priestia* sp. | EW1_B03 | OQ151833 | A, Bol, R | 1 | 1 | 1 | 1 | 0 | 1 | 1 | 1 | 1 | 1 | 1 |
| 6 | *Priestia* sp. | EW1_A04 | OQ151834 | A, Bol, R | 0 | 0 | 1 | 1 | 0 | 0 | 1 | 1 | 1 | 0 | 1 |
| 7 | *Priestia* sp. | EW1_C05 | OQ151835 | A, Bol, R | 0 | 1 | 1 | 1 | 1 | 1 | 1 | 1 | 1 | 1 | 1 |
| 8 | *Priestia* sp. | EW1_E05 | OQ151836 | A, Bol, R | 1 | 1 | 0 | 0 | 1 | 1 | 1 | 1 | 1 | 1 | 1 |
| 9 | *Pseudomonas* sp. | EW1_A06 | OQ151837 | A, Bol, R | 1 | 1 | 1 | 1 | 0 | 1 | 0 | 1 | 1 | 0 | 1 |
| 10 | *Priestia* sp. | EW1_B06 | OQ151838 | A, Bol, R | 1 | 1 | 0 | 1 | 1 | 1 | 1 | 1 | 1 | 1 | 1 |
| 11 | *Pseudomonas* sp. | EW1_E12 | OQ151839 | A, Bol, R | 1 | 1 | 0 | 1 | 1 | 1 | 0 | 1 | 1 | 1 | 1 |
| 12 | *Pseudomonas* sp. | EW2_H01 | OQ151840 | A, Bol, R | 1 | 1 | 0 | 0 | 0 | 1 | 0 | 1 | 1 | 1 | 1 |
| 13 | *Priestia* sp. | EW2_H10 | OQ151841 | A, Bol, R | 0 | 1 | 0 | 1 | 1 | 1 | 0 | 1 | 1 | 1 | 1 |
| 14 | *Paenibacillus* sp. | EW1_B01 | OQ151842 | A, Bol, RL | 1 | 1 | 0 | 0 | 0 | 1 | 0 | 1 | 1 | 1 | 1 |
| 15 | *Priestia* sp. | EW1_B02 | OQ151843 | A, Bol, RL | 1 | 1 | 1 | 1 | 0 | 1 | 1 | 1 | 1 | 1 | 1 |
| 16 | *Priestia* sp. | EW1_C02 | OQ151844 | A, Bol, RL | 1 | 1 | 1 | 1 | 0 | 1 | 1 | 1 | 1 | 1 | 1 |
| 17 | *Priestia* sp. | EW1_E02 | OQ151845 | A, Bol, RL | 1 | 1 | 1 | 1 | 0 | 0 | 1 | 1 | 1 | 1 | 1 |
| 18 | *Priestia* sp. | EW1_F03 | OQ151846 | A, Bol, RL | 1 | 1 | 1 | 1 | 0 | 1 | 1 | 1 | 1 | 1 | 1 |
| 19 | *Pseudomonas* sp. | EW1_G03 | OQ151847 | A, Bol, RL | 1 | 1 | 1 | 1 | 0 | 1 | 0 | 1 | 1 | 1 | 1 |
| 20 | *Bacillus* sp. | EW1_C04 | OQ151848 | A, Bol, RL | 1 | 1 | 1 | 1 | 1 | 1 | 0 | 1 | 1 | 1 | 1 |
| 21 | *Plantibacter* sp. | EW1_B05 | OQ151849 | A, Bol, RL | 0 | 1 | 1 | 1 | 1 | 1 | 0 | 1 | 1 | 1 | 1 |
| 22 | *Priestia* sp. | EW1_C06 | OQ151850 | A, Bol, RL | 1 | 1 | 1 | 1 | 1 | 1 | 0 | 1 | 1 | 1 | 1 |
| 23 | *Priestia* sp. | EW1_D06 | OQ151851 | A, Bol, RL | 1 | 1 | 1 | 1 | 1 | 1 | 1 | 1 | 1 | 1 | 1 |
| 24 | *Bacillus* sp. | EW1_H07 | OQ151852 | A, Bol, RL | 1 | 0 | 0 | 1 | 1 | 1 | 1 | 1 | 1 | 1 | 1 |
| 25 | *Plantibacter* sp. | EW1_F08 | OQ151853 | A, Bol, RL | 1 | 1 | 0 | 1 | 1 | 1 | 1 | 1 | 1 | 1 | 1 |
| 26 | *Stenotrophomonas* sp. | EW3_C08 | OQ151854 | A, Bol, RL | 1 | 1 | 1 | 1 | 1 | 1 | 0 | 1 | 1 | 1 | 1 |
| 27 | *Pseudomonas* sp. | EW1_E01 | OQ151855 | A, Bol, SL | 1 | 1 | 1 | 1 | 1 | 1 | 1 | 1 | 1 | 1 | 1 |
| 28 | *Priestia* sp. | EW1_H01 | OQ151856 | A, Bol, SL | 1 | 1 | 1 | 1 | 0 | 1 | 1 | 1 | 1 | 1 | 1 |
| 29 | *Bacillu*s sp. | EW1_H02 | OQ151857 | A, Bol, SL | 1 | 1 | 1 | 0 | 1 | 1 | 1 | 1 | 1 | 1 | 1 |
| 30 | *Pseudomonas* sp. | EW1_C03 | OQ151858 | A, Bol, SL | 1 | 1 | 0 | 1 | 0 | 1 | 0 | 1 | 1 | 1 | 1 |
| 31 | *Priestia* sp. | EW1_E03 | OQ151859 | A, Bol, SL | 1 | 1 | 1 | 1 | 0 | 1 | 0 | 1 | 1 | 1 | 1 |
| 32 | *Bacillus* sp. | EW1_B04 | OQ151860 | A, Bol, SL | 1 | 1 | 1 | 1 | 1 | 1 | 1 | 1 | 1 | 1 | 1 |
| 33 | *Priestia* sp. | EW1_E04 | OQ151861 | A, Bol, SL | 1 | 1 | 0 | 1 | 0 | 1 | 1 | 1 | 1 | 1 | 1 |
| 34 | *Frigoribacterium* sp. | EW1_F04 | OQ151862 | A, Bol, SL | 1 | 1 | 1 | 1 | 1 | 1 | 0 | 1 | 1 | 1 | 1 |
| 35 | *Priestia* sp. | EW1_H04 | OQ151863 | A, Bol, SL | 0 | 1 | 1 | 1 | 1 | 1 | 0 | 1 | 1 | 1 | 1 |
| 36 | *Bacillus* sp. | EW1_A05 | OQ151864 | A, Bol, SL | 1 | 1 | 1 | 1 | 1 | 1 | 0 | 1 | 1 | 1 | 1 |
| 37 | *Rhodococcus* sp. | EW1_D05 | OQ151865 | A, Bol, SL | 0 | 1 | 1 | 1 | 1 | 1 | 0 | 1 | 1 | 1 | 1 |
| 38 | *Rhodococcus* sp. | EW1_F05 | OQ151866 | A, Bol, SL | 1 | 1 | 1 | 1 | 0 | 1 | 0 | 1 | 0 | 1 | 1 |
| 39 | *Pseudomonas* sp. | EW1_E08 | OQ151867 | A, Bol, SL | 1 | 1 | 0 | 0 | 1 | 1 | 0 | 1 | 1 | 1 | 1 |
| 40 | *Pseudomonas* sp. | EW2_G07 | OQ151868 | A, Bol, SL | 1 | 1 | 0 | 1 | 1 | 1 | 1 | 1 | 1 | 1 | 1 |
| 41 | *Stenotrophomonas* sp. | EW3_G06 | OQ151869 | A, Bol, SL | 1 | 1 | 1 | 1 | 1 | 1 | 1 | 1 | 1 | 1 | 1 |
| 42 | *Pseudomonas* sp. | EW3_H08 | OQ151870 | A, Bol, SL | 1 | 1 | 0 | 1 | 1 | 1 | 0 | 1 | 1 | 1 | 1 |
| 43 | *Bacillus* sp. | EW1_A12 | OQ151871 | A, Buk, R | 0 | 0 | 1 | 1 | 1 | 1 | 0 | 0 | 1 | 1 | 1 |
| 44 | *Sphingomonas* sp. | EW2_A09 | OQ151872 | A, Buk, R | 0 | 1 | 0 | 1 | 1 | 1 | 0 | 1 | 1 | 1 | 1 |
| 45 | *Bacillus* sp. | EW3_F02 | OQ151873 | A, Buk, R | 1 | 1 | 1 | 1 | 1 | 1 | 0 | 1 | 1 | 1 | 1 |
| 46 | *Priestia* sp. | EW3_H02 | OQ151874 | A, Buk, R | 1 | 0 | 0 | 1 | 0 | 1 | 0 | 1 | 1 | 1 | 1 |
| 47 | *Stenotrophomonas* sp. | EW3_G04 | OQ151875 | A, Buk, R | 1 | 1 | 0 | 1 | 0 | 1 | 1 | 1 | 1 | 1 | 1 |
| 48 | *Priestia* sp. | EW1_H08 | OQ151876 | A, Buk, RL | 1 | 1 | 1 | 1 | 1 | 1 | 1 | 1 | 1 | 1 | 1 |
| 49 | *Sphingomonas* sp. | EW1_B09 | OQ151877 | A, Buk, RL | 1 | 1 | 0 | 1 | 1 | 1 | 0 | 1 | 0 | 1 | 1 |
| 50 | *Priestia* sp. | EW1_B10 | OQ151878 | A, Buk, RL | 1 | 1 | 1 | 1 | 1 | 1 | 0 | 1 | 1 | 1 | 1 |
| 51 | *Pseudomonas* sp. | EW2_C02 | OQ151879 | A, Buk, RL | 1 | 1 | 0 | 1 | 0 | 1 | 1 | 1 | 1 | 1 | 1 |
| 52 | *Priestia* sp. | EW2_E06 | OQ151880 | A, Buk, RL | 1 | 1 | 0 | 1 | 1 | 1 | 1 | 1 | 1 | 1 | 1 |
| 53 | *Micrococcus* sp. | EW2_B10 | OQ151881 | A, Buk, RL | 1 | 1 | 0 | 1 | 1 | 0 | 0 | 1 | 1 | 1 | 1 |
| 54 | *Stenotrophomonas* sp. | EW3_F05 | OQ151882 | A, Buk, RL | 1 | 1 | 1 | 1 | 1 | 1 | 0 | 1 | 1 | 1 | 1 |
| 55 | *Xanthomonas* sp. | EW3_H05 | OQ151883 | A, Buk, RL | 1 | 1 | 0 | 1 | 1 | 1 | 0 | 1 | 1 | 1 | 1 |
| 56 | *Priestia* sp. | EW1_A11 | OQ151884 | A, Buk, SL | 1 | 1 | 1 | 1 | 1 | 1 | 0 | 1 | 1 | 1 | 1 |
| 57 | *Brevibacillus* sp. | EW2_B06 | OQ151885 | A, Buk, SL | 1 | 1 | 0 | 1 | 1 | 1 | 0 | 1 | 1 | 1 | 1 |
| 58 | *Priestia* sp. | EW2_G06 | OQ151886 | A, Buk, SL | 1 | 1 | 0 | 1 | 1 | 1 | 1 | 1 | 1 | 1 | 1 |
| 59 | *Priestia* sp. | EW2_F09 | OQ151887 | A, Buk, SL | 1 | 1 | 0 | 1 | 1 | 1 | 0 | 1 | 1 | 1 | 1 |
| 60 | *Xanthomonas* sp. | EW2_F12 | OQ151888 | A, Buk, SL | 1 | 1 | 0 | 0 | 1 | 1 | 0 | 1 | 1 | 1 | 1 |
| 61 | *Plantibacter* sp. | EW3_A07 | OQ151889 | A, Buk, SL | 0 | 1 | 0 | 1 | 1 | 1 | 0 | 0 | 0 | 1 | 0 |
| 62 | *Priestia* sp. | EW1_G09 | OQ151890 | A, Bce, R | 1 | 1 | 1 | 1 | 1 | 0 | 0 | 1 | 1 | 1 | 1 |
| 63 | *Priestia* sp. | EW2_A07 | OQ151891 | A, Bce, R | 1 | 1 | 0 | 1 | 1 | 1 | 1 | 1 | 1 | 1 | 1 |
| 64 | *Brevibacillus* sp. | EW2_A08 | OQ151892 | A, Bce, R | 1 | 1 | 1 | 1 | 1 | 1 | 0 | 1 | 1 | 1 | 1 |
| 65 | *Priestia* sp. | EW2_B09 | OQ151893 | A, Bce, R | 1 | 1 | 1 | 1 | 1 | 1 | 1 | 1 | 1 | 1 | 1 |
| 66 | *Priestia* sp. | EW2_G09 | OQ151894 | A, Bce, R | 1 | 1 | 1 | 1 | 1 | 1 | 1 | 1 | 1 | 1 | 1 |
| 67 | *Priestia* sp. | EW2_G10 | OQ151895 | A, Bce, R | 1 | 1 | 1 | 1 | 1 | 1 | 1 | 1 | 1 | 1 | 1 |
| 68 | *Brevibacillus* sp. | EW2_C11 | OQ151896 | A, Bce, R | 1 | 1 | 0 | 1 | 0 | 0 | 0 | 1 | 1 | 1 | 1 |
| 69 | *Pseudomonas* sp. | EW3_F01 | OQ151897 | A, Bce, R | 1 | 1 | 1 | 1 | 1 | 1 | 1 | 1 | 1 | 1 | 1 |
| 70 | *Stenotrophomonas* sp. | EW3_F03 | OQ151898 | A, Bce, R | 1 | 1 | 0 | 1 | 1 | 1 | 1 | 1 | 1 | 1 | 1 |
| 71 | *Priestia* sp. | EW2_H07 | OQ151899 | A, Bce, RL | 1 | 1 | 1 | 1 | 1 | 0 | 0 | 1 | 1 | 1 | 1 |
| 72 | *Priestia* sp. | EW2_C09 | OQ151900 | A, Bce, RL | 1 | 1 | 0 | 1 | 1 | 1 | 1 | 1 | 1 | 1 | 1 |
| 73 | *Priestia* sp. | EW2_E09 | OQ151901 | A, Bce, RL | 1 | 1 | 0 | 1 | 1 | 1 | 1 | 1 | 1 | 1 | 1 |
| 74 | *Brevibacillus* sp. | EW2_H11 | OQ151902 | A, Bce, RL | 1 | 1 | 1 | 1 | 1 | 0 | 0 | 1 | 1 | 1 | 1 |
| 75 | *Stenotrophomonas* sp. | EW3_E04 | OQ151903 | A, Bce, RL | 1 | 1 | 1 | 1 | 0 | 1 | 1 | 1 | 1 | 1 | 1 |
| 76 | *Priestia* sp. | EW1_B11 | OQ151904 | A, Bce, SL | 0 | 1 | 1 | 1 | 1 | 1 | 1 | 1 | 1 | 1 | 1 |
| 77 | *Priestia* sp. | EW2_F03 | OQ151905 | A, Bce, SL | 1 | 0 | 1 | 1 | 0 | 1 | 1 | 1 | 1 | 1 | 1 |
| 78 | *Priestia* sp. | EW2_G03 | OQ151906 | A, Bce, SL | 1 | 1 | 1 | 1 | 1 | 1 | 0 | 1 | 1 | 1 | 1 |
| 79 | *Priestia* sp. | EW2_E05 | OQ151907 | A, Bce, SL | 1 | 1 | 0 | 1 | 1 | 1 | 1 | 0 | 1 | 1 | 1 |
| 80 | *Priestia* sp. | EW3_E03 | OQ151908 | A, Bce, SL | 0 | 0 | 0 | 0 | 0 | 1 | 1 | 1 | 1 | 1 | 1 |
| 81 | *Bacillus* sp. | EW1_C10 | OQ151909 | H, Bol, R | 0 | 1 | 0 | 1 | 1 | 1 | 0 | 1 | 1 | 1 | 1 |
| 82 | *Arthrobacter* sp. | EW3_E02 | OQ151910 | H, Bol, R | 1 | 1 | 0 | 1 | 0 | 0 | 1 | 0 | 1 | 1 | 1 |
| 83 | *Serratia* sp. | EW3_D04 | OQ151911 | H, Bol, R | 1 | 1 | 0 | 1 | 1 | 1 | 1 | 1 | 1 | 1 | 1 |
| 84 | *Pseudomonas* sp. | EW3_D06 | OQ151912 | H, Bol, R | 1 | 1 | 1 | 1 | 1 | 1 | 0 | 1 | 1 | 1 | 1 |
| 85 | *Bacillus* sp. | EW3_H06 | OQ151913 | H, Bol, R | 0 | 1 | 0 | 0 | 1 | 1 | 0 | 1 | 1 | 1 | 1 |
| 86 | *Bacillus* sp. | EW1_F10 | OQ151914 | H, Bol, RL | 1 | 1 | 0 | 0 | 0 | 1 | 1 | 1 | 1 | 1 | 1 |
| 87 | *Bacillus* sp. | EW1_C11 | OQ151915 | H, Bol, RL | 0 | 1 | 1 | 1 | 1 | 1 | 0 | 1 | 1 | 1 | 1 |
| 88 | *Bacillus* sp. | EW2_B02 | OQ151916 | H, Bol, RL | 1 | 1 | 1 | 1 | 1 | 1 | 1 | 1 | 1 | 1 | 1 |
| 89 | *Pseudomonas* sp. | EW2_H06 | OQ151917 | H, Bol, RL | 1 | 1 | 0 | 1 | 0 | 1 | 0 | 1 | 1 | 1 | 1 |
| 90 | *Bacillus* sp. | EW3_A01 | OQ151918 | H, Bol, RL | 0 | 1 | 0 | 1 | 0 | 1 | 0 | 1 | 1 | 1 | 1 |
| 91 | *Bacillus* sp. | EW3_F04 | OQ151919 | H, Bol, RL | 1 | 1 | 1 | 1 | 0 | 1 | 0 | 1 | 1 | 1 | 1 |
| 92 | *Bacillus* sp. | EW1_F09 | OQ151920 | H, Bol, SL | 1 | 1 | 0 | 1 | 1 | 1 | 0 | 1 | 1 | 1 | 1 |
| 93 | *Pseudomonas* sp. | EW2_G12 | OQ151921 | H, Bol, SL | 1 | 1 | 0 | 1 | 1 | 1 | 1 | 1 | 1 | 0 | 1 |
| 94 | *Bacillus* sp. | EW2_H12 | OQ151922 | H, Bol, SL | 1 | 1 | 1 | 1 | 1 | 1 | 1 | 1 | 1 | 1 | 1 |
| 95 | *Stenotrophomonas* sp. | EW3_G03 | OQ151923 | H, Bol, SL | 1 | 1 | 1 | 1 | 0 | 1 | 1 | 1 | 1 | 1 | 1 |
| 96 | *Stenotrophomonas* sp. | EW3_C05 | OQ151924 | H, Bol, SL | 1 | 1 | 0 | 1 | 1 | 1 | 0 | 1 | 1 | 1 | 1 |
| 97 | *Stenotrophomonas* sp. | EW3_C06 | OQ151925 | H, Bol, SL | 1 | 1 | 1 | 1 | 1 | 1 | 0 | 1 | 1 | 1 | 1 |
| 98 | *Pseudomonas* sp. | EW3_F06 | OQ151926 | H, Bol, SL | 1 | 1 | 0 | 1 | 1 | 1 | 1 | 1 | 1 | 1 | 1 |
| 99 | *Stenotrophomonas* sp. | EW3_B08 | OQ151927 | H, Bol, SL | 1 | 1 | 1 | 1 | 1 | 1 | 0 | 1 | 1 | 1 | 1 |
| 100 | *Enterobacter* sp. | EW3_G08 | OQ151928 | H, Bol, SL | 1 | 1 | 0 | 1 | 1 | 1 | 1 | 1 | 1 | 1 | 1 |
| 101 | *Bacillus* sp. | EW1_H05 | OQ151929 | H, Buk, R | 1 | 1 | 1 | 1 | 1 | 1 | 0 | 1 | 1 | 1 | 1 |
| 102 | *Priestia* sp. | EW1_B08 | OQ151930 | H, Buk, R | 0 | 1 | 1 | 1 | 1 | 1 | 1 | 1 | 1 | 1 | 1 |
| 103 | *Priestia* sp. | EW1_C08 | OQ151931 | H, Buk, R | 1 | 1 | 1 | 1 | 1 | 1 | 1 | 1 | 1 | 1 | 1 |
| 104 | *Priestia* sp. | EW2_A02 | OQ151932 | H, Buk, R | 0 | 1 | 0 | 1 | 1 | 1 | 0 | 1 | 1 | 1 | 1 |
| 105 | *Bacillus* sp. | EW3_A06 | OQ151933 | H, Buk, R | 1 | 1 | 0 | 1 | 1 | 0 | 0 | 1 | 1 | 1 | 1 |
| 106 | *Serratia* sp. | EW3_E06 | OQ151934 | H, Buk, R | 1 | 1 | 0 | 1 | 1 | 1 | 1 | 1 | 1 | 1 | 1 |
| 107 | *Enterobacter* sp. | EW3_B07 | OQ151935 | H, Buk, R | 1 | 1 | 0 | 1 | 1 | 1 | 0 | 1 | 1 | 1 | 1 |
| 108 | *Stenotrophomonas* sp. | EW3_G07 | OQ151936 | H, Buk, R | 1 | 1 | 0 | 1 | 1 | 1 | 1 | 1 | 1 | 1 | 1 |
| 109 | *Brevundimonas* sp. | EW1_D04 | OQ151937 | H, Buk, RL | 0 | 1 | 0 | 1 | 1 | 1 | 0 | 1 | 1 | 1 | 1 |
| 110 | *Sphingomonas* sp. | EW1_E10 | OQ151938 | H, Buk, RL | 0 | 1 | 0 | 1 | 1 | 1 | 0 | 1 | 1 | 1 | 1 |
| 111 | *Pseudomonas* sp. | EW1_H11 | OQ151939 | H, Buk, RL | 1 | 1 | 0 | 1 | 1 | 1 | 0 | 1 | 1 | 1 | 1 |
| 112 | *Priestia* sp. | EW2_E04 | OQ151940 | H, Buk, RL | 1 | 1 | 0 | 1 | 1 | 1 | 1 | 1 | 1 | 1 | 1 |
| 113 | *Neobacillus* sp. | EW2_C05 | OQ151941 | H, Buk, RL | 1 | 1 | 0 | 1 | 1 | 1 | 1 | 1 | 1 | 1 | 1 |
| 114 | *Bacillus* sp. | EW2_D05 | OQ151942 | H, Buk, RL | 1 | 1 | 0 | 1 | 1 | 1 | 1 | 1 | 1 | 1 | 1 |
| 115 | *Bacillus* sp. | EW2_B08 | OQ151943 | H, Buk, RL | 1 | 1 | 0 | 1 | 1 | 1 | 0 | 1 | 1 | 1 | 1 |
| 116 | *Brevibacillus* sp. | EW2_D11 | OQ151944 | H, Buk, RL | 1 | 1 | 0 | 1 | 0 | 1 | 0 | 1 | 1 | 1 | 1 |
| 117 | *Methylobacterium* sp. | EW3_D08 | OQ151945 | H, Buk, RL | 1 | 1 | 0 | 1 | 1 | 1 | 0 | 1 | 1 | 1 | 1 |
| 118 | *Pseudomonas* sp. | EW2_G02 | OQ151946 | H, Buk, SL | 1 | 1 | 1 | 1 | 0 | 1 | 0 | 1 | 1 | 1 | 1 |
| 119 | *Pseudomonas* sp. | EW2_A03 | OQ151947 | H, Buk, SL | 1 | 1 | 1 | 0 | 1 | 1 | 0 | 0 | 1 | 1 | 1 |
| 120 | *Brevibacillus* sp. | EW2_D04 | OQ151948 | H, Buk, SL | 1 | 1 | 0 | 1 | 1 | 1 | 1 | 1 | 1 | 1 | 1 |
| 121 | *Priestia* sp. | EW2_H04 | OQ151949 | H, Buk, SL | 1 | 0 | 1 | 1 | 0 | 1 | 0 | 1 | 1 | 1 | 1 |
| 122 | *Priestia* sp. | EW2_D09 | OQ151950 | H, Buk, SL | 1 | 1 | 0 | 1 | 1 | 1 | 1 | 1 | 1 | 1 | 1 |
| 123 | *Sphingomona*s sp. | EW2_D10 | OQ151951 | H, Buk, SL | 1 | 1 | 0 | 1 | 1 | 1 | 0 | 1 | 1 | 1 | 1 |
| 124 | *Bacillus* sp. | A1Bol | OQ151952 | A, Bol, RS | 0 | 1 | 0 | 1 | 1 | 0 | 0 | 1 | 0 | 1 | 1 |
| 125 | *Pseudomonas* sp. | A2Bol | OQ151953 | A, Bol, RS | 1 | 1 | 0 | 1 | 0 | 1 | 0 | 1 | 1 | 1 | 1 |
| 126 | *Lelliottia* sp. | A3Bol | OQ151954 | A, Bol, RS | 1 | 1 | 0 | 1 | 1 | 1 | 0 | 1 | 1 | 1 | 1 |
| 127 | *Stenotrophomonas* sp. | A4Bol | OQ151955 | A, Bol, RS | 1 | 1 | 0 | 1 | 1 | 1 | 1 | 1 | 1 | 1 | 1 |
| 128 | *Pseudomonas* sp. | A1Buk | OQ151956 | A, Buk, RS | 1 | 1 | 0 | 1 | 0 | 1 | 0 | 1 | 1 | 1 | 1 |
| 129 | *Pseudomonas* sp. | A2Buk | OQ151957 | A, Buk, RS | 1 | 1 | 1 | 1 | 1 | 1 | 1 | 1 | 1 | 1 | 1 |
| 130 | *Phytobacter* sp. | A3Buk | OQ151958 | A, Buk, RS | 1 | 1 | 0 | 1 | 1 | 1 | 1 | 1 | 1 | 1 | 1 |
| 131 | *Lelliottia* sp. | A4Buk | OQ151959 | A, Buk, RS | 1 | 1 | 0 | 1 | 1 | 1 | 1 | 0 | 1 | 1 | 1 |
| 132 | *Stenotrophomonas* sp. | A5Buk | OQ151960 | A, Buk, RS | 1 | 1 | 0 | 1 | 0 | 1 | 0 | 1 | 1 | 1 | 1 |
| 133 | *Pantoea* sp. | A1Bce | OQ151961 | A, Bce, RS | 1 | 1 | 0 | 1 | 1 | 1 | 1 | 1 | 1 | 1 | 1 |
| 134 | *Enterobacte*r sp. | A2Bce | OQ151962 | A, Bce, RS | 1 | 1 | 0 | 1 | 1 | 1 | 0 | 1 | 0 | 1 | 0 |
| 135 | *Pseudomonas* sp. | A3Bce | OQ151963 | A, Bce, RS | 1 | 1 | 0 | 1 | 1 | 1 | 1 | 1 | 1 | 1 | 1 |
| 136 | *Serratia* sp. | A4Bce | OQ151964 | A, Bce, RS | 1 | 1 | 0 | 1 | 1 | 1 | 1 | 1 | 0 | 1 | 0 |
| 137 | *Lelliottia* sp. | A5Bce | OQ151965 | A, Bce, RS | 1 | 1 | 0 | 1 | 1 | 1 | 1 | 0 | 1 | 1 | 1 |
| 138 | *Stenotrophomonas* sp. | A6Bce | OQ151966 | A, Bce, RS | 1 | 1 | 0 | 1 | 1 | 1 | 0 | 1 | 1 | 1 | 1 |
| 139 | *Pseudomonas* sp. | H1Bol | OQ151967 | H, Bol, RS | 1 | 1 | 0 | 1 | 1 | 1 | 0 | 1 | 1 | 1 | 1 |
| 140 | *Lelliottia* sp. | H2Bol | OQ151968 | H, Bol, RS | 1 | 1 | 0 | 1 | 1 | 1 | 0 | 1 | 0 | 1 | 1 |
| 141 | *Stenotrophomonas* sp. | H3Bol | OQ151969 | H, Bol, RS | 1 | 1 | 0 | 1 | 1 | 1 | 1 | 1 | 1 | 1 | 1 |
| 142 | *Stenotrophomonas* sp. | H1Buk | OQ151970 | H, Buk, RS | 1 | 1 | 0 | 1 | 0 | 1 | 0 | 1 | 1 | 1 | 1 |
| 143 | *Pseudomonas* sp. | H2Buk | OQ151971 | H, Buk, RS | 1 | 1 | 0 | 1 | 0 | 1 | 0 | 1 | 1 | 1 | 1 |
| 144 | *Lelliottia* sp. | H3Buk | OQ151972 | H, Buk, RS | 1 | 1 | 0 | 1 | 1 | 1 | 1 | 0 | 1 | 1 | 1 |

Table S2. Indole-3-acetic acid (IAA) concentration, 1-aminocyclopropane-1-carboxylate (ACC)-deaminase (ACCD) activity, and phosphate solubilization index of rhizosphere (RS) and endophytic bacteria from roots (R), rosette leaves (RL), and stem leaves (SL) of *A. halleri* and *A. arenosa* growing on Zn-Pb-Cd Bolesław (BOL) and Bukowno (BUK) waste heaps and on Bolestraszyce (BCE) reference area.

|  | IAA | | | ACCD | | | SI | | |
| --- | --- | --- | --- | --- | --- | --- | --- | --- | --- |
|  | ẋ ± SD | min (strain) | max (strain) | ẋ ± SD | min (strain) | max (strain) | ẋ ± SD | min (strain) | max (strain) |
| 1. *arenosa* BOL R | 28.50±7.41 | 18.40 (EW1_A04) | 44.14 (EW1_B06) | 0.25±0.14 | 0.08 (EW2_H01) | 0.48 (EW1_B03) | 2.30±0.84 | 1.0 (EW1_A03, EW1_A04) | 3.57 (EW1_E05) |
| *A. arenosa* BOL RL | 36.27±9.29 | 26.23 (EW1_F03) | 58.32 (EW1_B02) | 0.27±0.12 | 0.11 (EW1_F08) | 0.44 (EW1_B01) | 2.66±0.98 | 1.0 (EW1_E02) | 5.33 (EW1_C04) |
| *A. arenosa* BOL SL | 35.83±12.85 | 17.65 (EW1_H01) | 66.53 (EW3_H08) | 0.17±0.07 | 0.08 (EW1_H02) | 0.22 (EW1_F05) | 2.74±0.50 | 2.05 (EW1_H01) | 4.00 (EW3_H08) |
| *A. arenosa* BOL RS | 28.47±4.82 | 17.28 (EW2_A11) | 32.82 (EW3_C02) | 0.29±0.07 | 0.22 (EW2_E03, EW2_D08) | 0.40 (EW3_C02) | 3.48±2.82 | 1.0 (EW2_A11)) | 7.5 (EW1G_10) |
| *A. arenosa* BUK R | 31.55±12.37 | 16.90 (EW2_A09) | 43.40 (EW3_F02) | 0.21±0.06 | 0.14 (EW3_H02) | 0.28 (EW2_A09) | 2.56±1.43 | 1.5 (EW3_H02) | 5.0 (EW1_A12) |
| *A. arenosa* BUK RL | 36.87±8.00 | 27.35 (EW2_E06) | 48.62 (EW3_H05) | 0.16±0.05 | 0.11 (EW2_B10) | 0.25 (EW2_C02) | 2.12±0.90 | 1.0 (EW2_B10) | 4.0 (EW3_F05) |
| *A. arenosa* BUK SL | 30.77±7.63 | 21.38 (EW1_A11) | 40.04 (EW3_A07) | 0.20±0.15 | 0.10 (EW2_F09) | 0.48 (EW1_A11) | 2.85±1.32 | 2.0 (EW1_A11) | 5.5 (EW2_F09) |
| *A. arenosa* BUK RS | 27.97±3.76 | 23.99 (EW1_H09) | 31.46 (EW1_A10) | 0.21±0.05 | 1.15 (EW1_H09) | 0.24 (EW1_A10) | 3.13±1.31 | 2.0 (EW2_E01) | 4.63 (EW3_D02) |
| *A. arenosa* BCE R | 52.26±24.28 | 23.62 (EW2_C11) | 87.05 (EW2_G09) | 0.14±0.06 | 0.11 (EW2_G10) | 0.23 (EW2_C11) | 2.61±1.42 | 1.0 (EW1_G09, EW2_C11) | 5.2 (EW2_G09) |
| *A. arenosa* BCE RL | 39.04±5.44 | 33.32 (EW2_E09) | 44.14 (EW2_C09) | 0.11±0.01 | 0.11 (EW2_E09) | 0.12 (EW2_H07) | 1.76±0.72 | 1.0 (EW2_H07, EW2_H11) | 2.56 (EW2_C09) |
| *A. arenosa* BCE SL | 27.26±4.24 | 23.25 (EW1_B11) | 31.46 (EW2_G03) | 0.18±0.13 | 0.09 (EW2_E05) | 0.38 (EW1_B11) | 1.95±0.44 | 1.5 (EW1_B11) | 2.56 (EW2_E05) |
| *A. arenosa* BCE RS | 32.18±4.82 | 22.87 (EW2_C08) | 37.48 (EW2_F10) | 0.16±0.05 | 0.13 (EW2_C08) | 0.25 (EW2_C06) | 3.86±2.43 | 2.29 (EW2_C06) | 8.50 (EW2_F10) |
| *A. halleri* BOL R | 56.83±16.08 | 44.89 (EW3_E02) | 75.11 (EW3_H06) | 0.19±0.11 | 0.10 (EW3_H06) | 0.32 (EW1_C10) | 2.38±0.80 | 1.0 (EW3_E02) | 2.8 (EW1_C10) |
| *A.* *halleri* BOL RL | 32.35±13.08 | 20.26 (EW3_A01) | 54.22 (EW3_F04) | 0.30±0.19 | 0.09 (EW2_H06) | 0.56 (EW3_A01) | 2.48±1.48 | 1.3 (EW3_A01) | 5.0 (EW1_F10) |
| *A. halleri* BOL SL | 96.21±68.26 | 31.08 (EW1_F09) | 185.93 (EW2_H12) | 0.17±0.04 | 0.12 (EW3_F06) | 0.21 (EW3_C05) | 2.52±0.40 | 2.07 (EW1_F09) | 3.4 (EW3_C05) |
| *A. halleri* BOL RS | 29.91±4.80 | 23.99 (EW2_E10) | 31.46 (EW2_C02) | 0.22±0.08 | 0.19 (EW2_E10) | 0.25 (EW2_C02) | 2.0±0.40 | 1.71 (EW2_C02) | 2.29 (EW2_E08) |
| *A. halleri* BUK R | 103.56±78.24 | 18.40 (EW2_A02) | 237.05 (EW3_E06) | 0.22±0.12 | 0.07 (EW1_H05) | 0.48 (EW2_A02) | 2.20±0.80 | 1.0 (EW3_A06) | 3.57 (EW3_B07) |
| *A. halleri* BUK RL | 29.71±6.61 | 25.86 (EW2_E04, EW2_C05) | 43.02 (EW2_D11) | 0.14±0.05 | 0.11 (EW2_B08, EW2_E04) | 0.21 (EW2_C05) | 3.33±2.26 | 2.0 (EW1_B08, EW1_D04) | 9.0 (EW2_D11) |
| *A. halleri* BUK SL | 29.51±6.86 | 19.14 (EW2_H04) | 37.43 (EW2_D10) | 0.10±0.02 | 0.09 (EW2_G02) | 0.13 (EW2_H04) | 2.38±0.23 | 2.09 (EW2_D09) | 2.6 (EW2_A03, EW2_G02) |
| *A. halleri* BUK RS | 29.97±18.0 | 31.46 (EW1_A10) | 23.99 (EW1_H09) | 0.17±0.80 | 0.15 (EW1_H09) | 0.24 (EW1_A10) | 3.80±1.71 | 2.29 (EW1_A10) | 4.63 (EW3_D02) |

Table S3. Parameters and conditions of the metal concentration analysis with the usage of inductively coupled plasma mass spectrometry (ICP-MS) method and device ICP-MS 2030, Shimadzu, Japan

| Parameters | Values |
| --- | --- |
| plasma gas flow rate | 8.0 L × min^-1^ |
| auxiliary gas flow rate | 1.1 L × min^-1^ |
| nebulization gas flow rate | 0.6 L × min^-1^ |
| nebulizer | coaxial |
| chamber | cyclone (glass) |
| spray chamber temperature | 3°C |
| drain | gravity fed |
| internal standard | automatic addition |
| sampling depth | 5 mm |
| collision cell gas flow (He) | 6 mL × min^-1^ |
| cell voltage | 21V |
| energy filter | 7.0V |
